# Deep imagination is a close to optimal policy for planning in large decision trees under limited resources

**DOI:** 10.1038/s41598-022-13862-2

**Published:** 2022-06-21

**Authors:** Chiara Mastrogiuseppe, Rubén Moreno-Bote

**Affiliations:** 1 Department of Information and Communication Technologies, Center for Brain and Cognition, Universitat Pompeu Fabra, Barcelona, Spain; 2 Serra Húnter Fellow Programme, Universitat Pompeu Fabra, Barcelona, Spain

**Keywords:** Decision, Computer science

## Abstract

Many decisions involve choosing an uncertain course of action in deep and wide decision trees, as when we plan to visit an exotic country for vacation. In these cases, exhaustive search for the best sequence of actions is not tractable due to the large number of possibilities and limited time or computational resources available to make the decision. Therefore, planning agents need to balance breadth—considering many actions in the first few tree levels—and depth—considering many levels but few actions in each of them—to allocate optimally their finite search capacity. We provide efficient analytical solutions and numerical analysis to the problem of allocating finite sampling capacity in one shot to infinitely large decision trees, both in the time discounted and undiscounted cases. We find that in general the optimal policy is to allocate few samples per level so that deep levels can be reached, thus favoring depth over breadth search. In contrast, in poor environments and at low capacity, it is best to broadly sample branches at the cost of not sampling deeply, although this policy is marginally better than deep allocations. Our results can provide a theoretical foundation for why human reasoning is pervaded by imagination-based processes.

## Introduction

When we plan our next holiday trip, we decide on a course of action that has a tree structure: first, choose a country to visit, then the city to stay in, what restaurant to go to, and so on. Planning is a daunting problem because the number of scenarios that could be considered grows rapidly with the depth and width of the associated decision tree. As we are limited by the amount of available time, number of neurons or energy to mentally simulate the best plan^1–6^, the dilemma that arises then is how to allocate limited search resources over large decision trees. Should we consider many countries for our next vacation (breadth) at the cost of not evaluating very thoroughly any of them, or should we consider very few countries more deeply (depth) at the risk of missing the most exciting one? The above problem is one example of the so-called *breadth-depth* (BD) dilemma, important in tree search algorithms^7,8^, optimizing menu designs^9^, decision-making^4,10,11^, knowledge management^12^ and education^13^.

Algorithms that look for the best course of action in large decision trees rarely make explicit the limited resources that are available, and thus are ignorant of BD tradeoffs. For instance, standard dynamic programming techniques estimate the value of all tree nodes simultaneously^14^ and Monte Carlo tree search^15^ approximate state values by efficiently exploring and expanding promising tree nodes. These methods guarantee optimality if all states and actions are sampled with probability one on the long run. However, in extremely large problems, like in infinitely many-armed bandits^4,10,16,17^ or in meta-reasoning approaches with vast action-computation spaces^1,18–20^, exhaustive exploration of all actions and states an enough number of times is not under reach under limited resources. The problem that arises then is how many actions and states should be ignored for planning.

Optimization of BD tradeoffs have been studied using the framework of infinitely many-armed bandits and combinatorial multi-armed bandits where resources can be arbitrarily allocated among many options. These include one-shot infinitely many-armed Bernoulli^4^ and Gaussian^10^ bandits with compound actions, sequential infinitely many-armed Bernoulli bandits^16^ and broader families thereof^17^ with simple actions, and sequential combinatorial multi-armed bandits with compound actions^21^. These studies show that, even for unbounded resources, it is optimal to ignore the vast majority of options to focus sampling on a relatively small number of them that sublinearly scales with capacity^4,10^. However, the described optimal BD tradeoffs have been limited to trees of depth one, and in most cases results are valid only asymptotically as search capacity goes to infinity. Therefore, how to optimally balance breadth and depth search in decision trees remains an unresolved problem.

In this paper we characterize the optimal policies for the allocation of finite search capacity over an infinitely large decision tree (Fig. 1). We consider ensembles of decisions trees with a random structure of rewards. Thus, by describing optimal allocation policies that are not tied to any particular structure, we expect that the discovered features of the policies are of general validity. In our model, the immediate rewards that would result from actually visiting the tree nodes have an unknown expectation that can be learned by sampling them through e.g. mental simulation. However, due to the finite number of samples available, called *capacity*, the agent needs to determine the best way to allocate them over the nodes of the tree. The agent can allocate samples to simulate many short courses of action (breadth search, Fig. 1a) at the risk of not evaluating any of them deeply, or can allocate samples to simulate few long courses of action (depth search) at the risk of missing the most relevant ones. We consider the problem of allocating samples simultaneously in one shot without knowing their individual outcomes. One-shot allocations describe situations where the dispatching of sampling resources needs to be made before feedback is received, and thus they are good models when the delays in the feedback are longer than the time needed to allocate the resources, like when assigning budget to research or vaccine projects. While selecting the most promising course of action once samples have been allocated and observed is an easy selection problem, finding the one-shot sampling policy that maximizes the expected value of the most promising course is a harder combinatorial problem.

We describe the optimal sampling policy over infinitely large decision trees as a function of the capacity of the agent and the difficulty of obtaining rewards, in both the time discounted and undiscounted cases. Exploiting symmetries, we develop an efficient *diffusion-maximization* algorithm for the exact evaluation of the search policies with computational cost of order ${\mathcal {O}}(bd^2)$, where *d* is the number of sampled levels of the decision tree and *b* is the sampling branching factor, much better than the scaling ${\mathcal {O}}(b^d)$ using backward induction on the tree itself. We find that it is generally better to sample very deeply the decision tree such that information over many levels can be gathered, a policy that we call *deep imagination*, in analogy to how human imagination works^22–27^.

## Results

**Figure 1:**
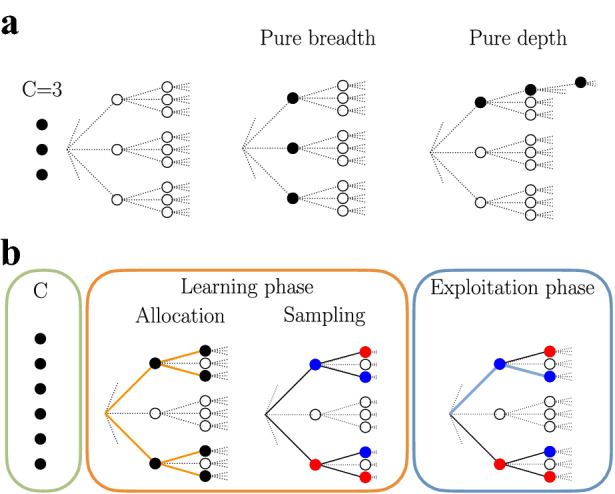
Planning decisions in large decision trees with finite sampling capacity. (**a**) Breadth-depth dilemma in an infinitely large decision tree. Nodes correspond to states, and edges correspond to possible actions resulting in deterministic transitions from the parent node to the selected children node. Sampling a node results in learning whether the node would promise high or low expected reward when actually visiting it. The agent can allocate finite sampling capacity *C* ($C=3$ in the example) to gain information about the structure of expected rewards. Samples can be allocated broadly in the first levels (breadth search, middle panel), deeply in few branches (depth search, right panel), or using any intermediate policy. (**b**) The agent solves the planning problem in two phases: in the learning phase (orange panel), samples are allocated in one shot to learn about the magnitudes of the expected rewards of the nodes, and in the exploitation phase (blue panel) the learned expected rewards are used to select the optimal path (blue path). In the example, the 6 samples are allocated (allocation; closed circles), after which the agent learns about the expected rewards from the sampled nodes (sampling; blue, positive expected reward $R_+$ learnt; red, negative expected reward $R_-$ learnt). For the case $p=\frac{1}{2}$, illustrated in the figure, the expected rewards take values $R_+=+1$ and $R_-=-1$ with equal probability, while the expected rewards for unsampled nodes remain 0, and are not indicated in the figure. After sampling, the agent can select the optimal sequence of actions, the one with the highest expected cumulative reward, which in this case corresponds to the blue path, with expected cumulative reward equal to $1+1=2$.

### A model for search in wide and deep decision trees with finite capacity

We consider a Markov Decision Process (MDP) over a large decision tree (Fig. 1; see Methods Sect. “Model details” for more details). The underlying structure is a directed rooted tree with infinite depth and infinite branching factor. Each path leaving the root node can be seen as a possible course of action that could be followed by the agent. Actions correspond to edges, which lead to deterministic transitions between selected nodes. However, before acting, the agent needs to learn the reward structure of the tree as well as possible, as they do not know whether visiting a node promises high or low expected immediate reward. If the agent had infinite search capacity, they could sample all nodes and thus select the best path through them. However, an agent with limited capacity can only sample a finite number of nodes and base their decision on the observed quality of the sampled nodes alone. Sampling a node can be viewed as the ‘mental simulation’ of visiting such a node, which results in updating the expected immediate reward that would result from actually visiting it in the future. We assume that the agent has a correct model of the tree structure, possible transitions, and probabilities of having nodes with different expected rewards, but before sampling does not know the actual expected values of the rewards. Therefore, the central problem is how to allocate finite sampling capacity to maximize expected cumulative reward over the best possible course of action.

More specifically, we consider an agent that has two sets of available actions divided into two different phases, a learning and an exploitation phase. In the learning phase, the agent has a finite number *C* of samples to be allocated over the nodes of the decision tree (Fig. 1b). In this phase, we distinguish between the state of knowledge that the agent has before and after sampling a node regarding the expected reward that would result from visiting that node. Before sampling the node, the agent knows that visiting it would result in an expected reward equal to zero, to model their initial state of ignorance. After sampling the node, their knowledge about what would be the expected reward if visiting it in the future changes. It can either move to a high expected reward $R_+=1$ with probability *p*, or move to a low expected reward $R_-=-\frac{p}{1-p}$ with probability $1-p$, identically and independently for each sampled node. Note that neither before nor during sampling a node the agent actually visits that node. Indeed, we think of the sampling process as an internal mental process of the agent that simulates experiences before actually acting on the world^28,29^. Importantly, the choice for the sizes and probabilities of the binary expected rewards is made without loss of generality to satisfy the *zero-average* constraint $p\cdot R_++(1-p)\cdot R_-=0$. In this way the total expectation of a sampled node equals zero, the same as the expectation of a non-sampled node, consistent with the agent correctly knowing the reward structure of the problem.

The probability *p* that sampling a node changes the agent’s knowledge state to a high expected reward defines the easiness of finding rewards in the future, with high *p* corresponding to a ‘rich’ environment, and low *p* corresponding to a ‘poor’ environment. When *p* becomes very small, positive expected rewards are rare and have an unpredictable structure over the tree, similar to the situations where rewards are very sparse and have a somehow complex structure.

The agent allocates *C* samples over an equal number of nodes in the tree with the aim of learning its expected reward structure. As a result, the knowledge about the expected rewards of sampled nodes is updated as explained above, with some of the nodes moving to expected reward $R_+$ and some others to $R_-$, independently. Non-sampled nodes remain having expected reward equal to 0. We assume that the allocation of the *C* samples is made simultaneously, in ‘one-shot’, and thus it cannot use feedback from the knowledge updates of other sampled nodes. This is a reasonable assumption when feedback delays are larger than the available time to allocate resources, as it happens in many common situations^4,10^.

The exploitation phase is more straightforward: based on the expected rewards for each tree node *s*, $R(s) \in \{R_+,R_-,0\}$, that have been learnt in the first phase, the agent selects the path with the highest expected cumulative reward (Fig. 1b, right panel). In principle, this should be done by using the Bellman equation over the infinitely large decision tree by including all sampled and non-sampled nodes, where we have *C* sampled nodes and an infinite number of non-sampled nodes. Fortunately, one can restrict the Bellman equation to only the set of nodes that connect sampled nodes to the root node, making tractable the solution of the problem since the resulting sub-tree is finite. We ignore the possibility of choosing a path with all nodes not being sampled, which will have expected cumulative reward equal to zero. Note, however, that the optimal path can traverse non-sampled nodes if necessary. Finally, the goal of the agent is to find the optimal allocation of samples, which is the one that maximizes the expected cumulative reward of the best path over all possible allocation policies. Finding the optimal allocation policy is a hard combinatorial search that is not tractable in general, and thus we restrict our analysis below to some rich allocation families.

We remark here that the zero-average constraint is both convenient and necessary. By enforcing it, a random path of any length over the tree has expected cumulative reward equal to zero. Therefore, positive expected cumulative rewards inferred in the exploitation phase are relative to random strategies that are ignorant of the learning phase. More importantly, we consider below allocation families where the probability of sampling nodes in a level can be smaller than one. If expected immediate reward before sampling were positive, then the optimal strategy would be to assign zero sampling probability to every node so that sampling capacity is never exhausted. This strategy will promise unbounded expected cumulative reward in the time undiscounted case. In contrast, with the zero-average constraint, unbounded reward is not possible as sampling is necessary to learn which nodes have a positive expected reward.

In our model, rewards are independently and identically distributed among nodes; the path with highest expected accumulated (discounted or not) reward is the one chosen. Our framework differs from many optimization algorithms where rewards are found only at the leaf nodes^15,30,31^. By letting the agent accumulate the outcomes of the nodes in a path, we model real-life decisions where multiple levels of the tree must be evaluated and contribute to the total reward. A relevant example of the above model is holiday planning: in the first level of the decision tree an agent can choose one out of many different countries, from where they can choose one of many different cities, and so on. How satisfactory the trip depends on how positively the country, the city and the elements in the different levels will be evaluated. The modeled planning process can be divided into two phases. In the learning phase, the agent learns about what cities, museums and such would be more desirable. Here, actions do not correspond to actually visiting the nodes of the tree, but to observations or mental simulations thereof that are limited in amount and are planned beforehand. These observations (e.g., reading books) or mental simulations (e.g., memory recollections) change the belief that the agent has about the expected reward that would result from actually visiting the nodes in the future. This knowledge is used in the exploitation phase to design the best course of action before the holiday trip commences. The hardest problem is to optimally allocate a finite search resource over the vast decision tree.

### Value computation and optimal sample allocations

**Figure 2:**
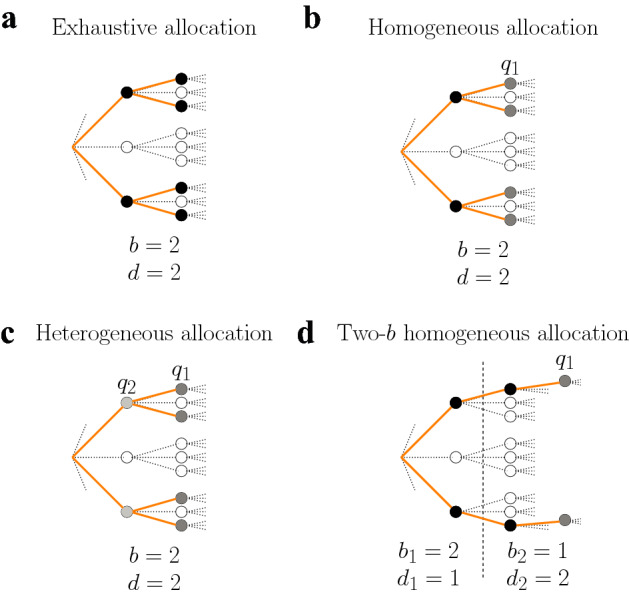
Families of allocation policies to sample an infinite decision tree. (**a**) In exhaustive allocation, the agent fully samples (black dots) the nodes with chosen branching factor *b* and tree depth *d*. (**b**) In homogeneous allocation, the agent chooses the branching factor *b* and samples as deep as resources allow. The first $d-1$ levels of the tree will be allocated with probability one (black dots), while the sampling probability of the last level $q_1 \le 1$ (grey dots) is chosen such that the average capacity constraint in Eq. (6) is satisfied. (**c**) In heterogeneous allocation, the agent is free to choose the branching factor *b* and the probability $q_{d-l+1}$ of sampling the nodes (grey dots) at the $l-th$ level (note reversed order of index). Nodes in the same level share the same probabilities of being sampled. Sampling probabilities are chosen such that the average capacity constraint in Eq. (6) is satisfied. (**d**) In two-*b* homogeneous allocation, the agent samples the first $d_1$ levels of the tree with branching factor $b_1$ and the following $d_2$ levels with $b_2$. As in homogeneous allocations, only nodes in the last level of the tree are allocated with non-one probability $q_1$ (grey dots) such that the average capacity constraint in Eq. (10) is satisfied.

We first introduce *exhaustive* allocation policies (Fig. 2a), which sample all nodes of a decision tree of depth *d* and branching factor *b*. With this policy a finite sub-tree is fully sampled within the initial infinite decision tree. We then introduce *selective* allocation policies (Fig. 2b,c), which allow the agent to select *b* and also the probability of drawing samples at each tree level under the constraint that the number of allocated samples is on average a fixed capacity *C*. Finally, we introduce *two-branching (two-b) factors* allocation policies (Fig. 2d), which allow the agent to sample the tree with a different branching factor in superficial and deep levels. As we show below, the above policies are rich enough to display a broad range of behaviors. For each policy we show how to compute its value, defined as the expected cumulative reward of the optimal path. We first consider the undiscounted case, and later we generalize our results to the discounted setting. To avoid cluttered text, we refer to *expected rewards* simply as *rewards*, but the reader should bear in mind that samples change the knowledge about the expected reward of visiting the node in the future.

#### Exhaustive allocation

An exhaustive allocation policy fully samples all the nodes of a tree with depth *d* and branching factor *b* identical for every node. Here, we first compute the probability that an agent can find a path with cumulative reward equal to the depth *d* in such a tree (remember the omission of ‘expected’ from now on). After this, we calculate the value, $V_{b,d}$, of playing such a tree to develop a useful tool.

We first show that, in general, it is not possible to find a path with all nodes having a positive reward. Hence, an optimal path is likely to find a blocked node, that is, a node where all possible actions lead to negative reward, and thus extreme optimism cannot be guaranteed. By assuming that the reward in a node has value $R_+=1$ with probability *p* and setting $R_-$ (which is negative) such that the zero-average constraint is satisfied, then the event of finding a path with all positive rewards corresponds to the event that the cumulative reward of the optimal path is the depth *d* of the tree. We denote the cumulative reward of the optimal path in a tree of depth *d* by $J_d$, and thus we ask for the probability $P(J_d=d)$. If the tree has depth $d=1$ and branching factor *b*, then $P(J_1=1) = 1 - (1-p)^b$. This expression follows from the fact that there are *b* possible actions, and the probability that none of those actions leads to a reward equal to $R=1$, and thus it is blocked, is $(1-p)^b$.

For $d>1$ we make use of the quantity $Q_{d}=R_{d}+J_{d-1}$, known as action-value, defined as the cumulative reward obtained by first choosing one of the *b* branches and collect immediate reward $R_d$, and then choosing the best sequence of branches in the remaining $d-1$ levels to collect cumulative reward $J_{d-1}$. Note that in principle there are *b* different action-values $Q_{d}$, one per branch, but as all of them are statistically indistinguishable, an index is not made explicit (the same happens for the rewards $R_{d}$). Using this relationship we find

1 $$\begin{aligned} P(J_{d}=d)=1 - \left( 1-P(Q_{d}=d)\right) ^b = 1-\left( 1-pP(J_{d-1}=d-1)\right) ^b ,\; d > 1 . \end{aligned}$$

The first equality in Eq. (1) comes from the fact that to get a cumulative reward $J_d<d$ it is necessary that none of the *b* possible actions from the root node leads to $Q_{d}=d$, and that each of those events are statistically independent. The second equality comes from the fact that $P(Q_{d} = d)= pP(J_{d-1}=d-1)$, which is the probability that a particular action from the root node is followed by a state with $R_d=1$, which has probability *p*, and afterward followed by an optimal path with cumulative reward $d-1$, which has probability $P(J_{d-1}=d-1)$.

We can use the above expression to find cases where the probability of having optimal paths with cumulative reward *d* approaches zero as *d* increases. For $b=2$ and $p=\frac{1}{2}$, using Eq. (1) we obtain $P(J_1=1) = \frac{3}{4}$ and $P(J_{d}=d) = 1 - \left( 1 - \frac{1}{2} P(J_{d-1}=d-1) \right) ^2$ for $d > 1$. We see that $\lim _{d \rightarrow \infty } P(J_d=d)= 0$, as the only solution to the fixed point equation $P=1-(1-P/2)^2$ is $P=0$. Therefore, the probability that the agent finds a blocking node is one as the tree depth increases. For any positive integer *b* and $p \in [0,1]$, the fixed point equation for large *d* becomes $1-P = (1-pP)^b$. As the rhs is convex in *P*, positive and has its maximum at $P=0$, the fixed point equation has a non-zero solution only when the rhs’ slope at the origin is smaller than $-1$, that is, when $pb>1$. Therefore, if *p* decreases, then a large enough *b* ensures a non-zero probability of finding an optimal path with cumulative reward equal to the tree depth. In contrast, if $b \le p^{-1}$, then the probability that the path is blocked with nodes having negative rewards is one.

**Figure 3:**
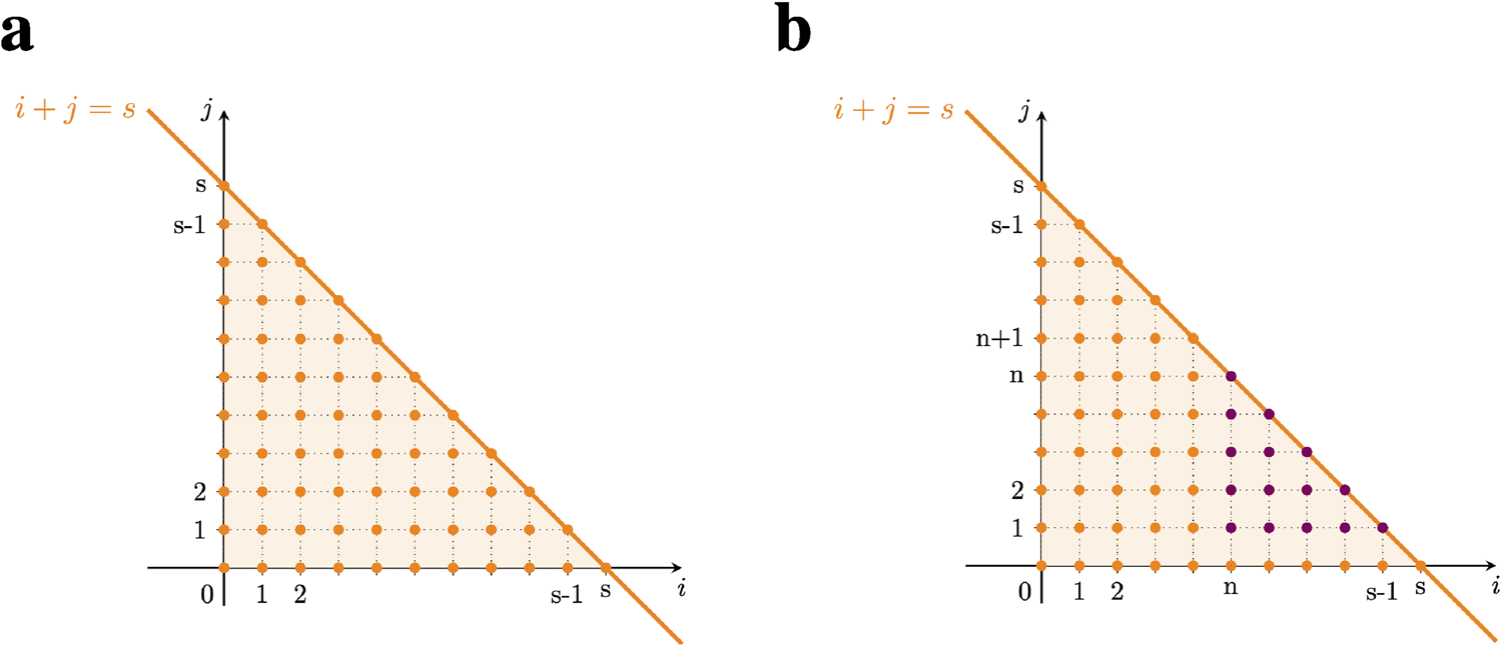
Insights on the algorithmic complexity. For rational values of $p=\frac{1}{n+1}$ and $p=\frac{n}{n+1}$, with integer *n*, the possible state values $J_s$ at level *s* are $k=i-nj$, with *i* and *j* respectively the number of times the positive and negative reward is observed, such that $i,j \ge 0$ and $i+j \le s$. For different values of *i* and *j* within the allowed set, *k* can have repeated values leading to degenerate states. (**a**) Possible (*i*, *j*) pairs for admissible states at level *s*. (**b**) Same as in (a); in purple, the pairs leading to an already considered state (overlapping states). A detailed description of the algorithmic complexity, together with the more general analytical solutions, can be found in Sect. “Value of exhaustive or selective search in a large tree with rational* p*” of the Methods.

After establishing that extreme optimism is not always guaranteed, we turn to the problem of finding the value of playing the tree with *d* levels and branching factor *b*, defined as the expected cumulative reward of the optimal paths over such a tree. We provide here the analytical solution for $p=\frac{1}{2}$. The more general analytical solutions for the rational cases of $p=\frac{1}{n+1}$ and $p=\frac{n}{n+1}$ with *n* a positive integer are described in Sect. “Value of exhaustive or selective search in a large tree with rational* p*” of the Methods, together with a discussion of the algorithmic complexity (see Fig. 3 for a graphical insight).

For simplicity and without loss of generality we set $R(s)=R_+=1$ and $R(s)=R_-=-1$ with probabilities $p=\frac{1}{2}$, which satisfies the zero-average constraint. Thus, the cumulative reward of a path following a sequence of actions through the tree with *d* levels can take values $J_d \in \{-d, -d+2,\ldots , d-2, d\}$. The size of this set is order ${\mathcal {O}}(d)$, which allows us to compute the value of any tree of depth *d* in polynomial time. We first compute the probability $P(J_1)$ of the value $J_1 \in \{-1,1\}$ of playing a tree of depth 1, and then compute the probability $P(J_{d})$ of the value $J_d$ of playing a tree of depth *d* recursively from $P(J_{d-1})$. Above we showed that $P(J_1=1) = 1 - P(J_1=-1) = 1 - 2^{-b}$ for a tree of depth 1. Thus, the value of playing such a tree is the average of $J_1$ over sampling outcomes, which equals $V_1 = {\mathbb {E}}(J_1) = 1 - 2^{1-b}$.

Our algorithm is based on alternating *diffusion* and *maximization* steps as follows. To find the probability $P(J_{d})$ from $P(J_{d-1})$, we first remind that the action-value $Q_{d}$ is defined as the cumulative reward by taking one action at the root, collect reward $R_d$ and then follow the optimal path in a tree with $d-1$ levels. Written as $Q_{d}=R_d+J_{d-1}$, it has probabilities

2 $$\begin{aligned}&P(Q_{d}=d) = \frac{1}{2} P(J_{d-1}=d-1)\nonumber \\&P(Q_{d}=d-2) = \frac{1}{2} P(J_{d-1}=d-1) + \frac{1}{2} P(J_{d-1}=d-3)\nonumber \\&\vdots \\&P(Q_{d}=2-d) = \frac{1}{2} P(J_{d-1}=3-d) + \frac{1}{2} P(J_{d-1}=1-d)\nonumber \\&P(Q_{d}=-d) = \frac{1}{2} P(J_{d-1}=1-d) \;.\nonumber \end{aligned}$$

This mapping from $P(J_{d-1})$ to $P(Q_{d})$ is a *diffusion* step, as the state $J_{d-1}=k$ diffuses to higher, $k+1$, and lower, $k-1$, states of $Q_{d}$ with probability $p=\frac{1}{2}$. We recognize the first identity in Eq. (2) as the probability that a chosen action followed by the optimal path over a tree with $d-1$ levels leads to a cumulative reward *d* for the case $p=\frac{1}{2}$, as discussed above.

The diffusion step is followed by the *maximization* step, which maps $P(Q_{d})$ into $P(J_{d})$ by

3 $$\begin{aligned} P(J_{d}=k) = P(Q_{d} \le k)^b - P(Q_{d} \le k-1)^b, \end{aligned}$$

for $k \in \{-d, -d+2,\ldots , d-2, d \}$. Eq. (3) represents a maximization step because the agent will choose the best action out of *b* available actions, and it expresses that the probability of $J_d=k$ equals the probability of finding at least one action with at most a value of $Q_d=k$.

In summary, iterating the diffusion and maximization steps in Eqs. (2,3) with initial conditions $P(J_1=1) = 1-P(J_1=-1) = 1 - 2^{-b}$ allows us to compute the value of playing a tree with *d* levels and *b* branches by $V_{d,b} = {\mathbb {E}}(J_{d})$. The number of operations required to determine the value of such a tree is ${\mathcal {O}}(bd^2)$, as the diffusion step requires ${\mathcal {O}}(d^2)$ operations due to the presence of *d* levels and ${\mathcal {O}}(d)$ different states at each level, and the maximization step involves ${\mathcal {O}}(b)$ operations for each $J_d=k$ in the calculation of *b*-th powers. In contrast, a direct solution to the problem using dynamic programming without exploiting symmetries requires ${\mathcal {O}}(b^d)$ operations. This is because the complexity is dominated by the number of nodes in the level before the last one, where there are $b^{d-1}$ nodes, and *b* operations are needed in each one to solve the *max* operator before implementing backward induction. In addition, the complexity of dynamic programming does not take into account the additional need to average over the samples’ outcomes, while the diffusion-maximization method in Eqs. (2,3) provides the exact expected value of playing the tree.

**Figure 4:**
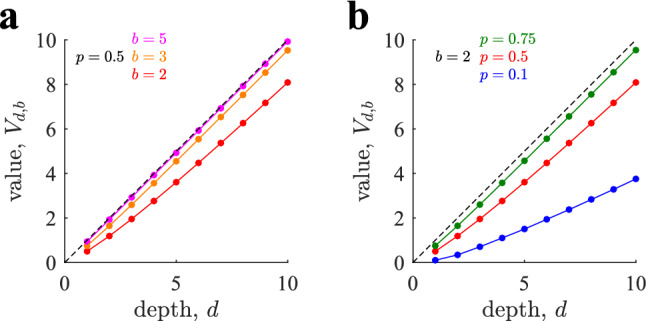
Value $V_{d,b}$ of playing a tree of depth *d* and branching factor *b* with exhaustive sampling. (**a**) The value (expected cumulative reward) of playing a tree increases monotonically with both its depth *d* and its branching factor *b*. In all cases $p=\frac{1}{2}$. For $b=5$ (pink) the value is very close to the maximum possible value (dashed, diagonal line). (**b**) The value of playing the tree grows with the probability *p* of high expected reward in their nodes. In all cases $b=2$. In both panels, lines correspond to analytical predictions from the diffusion-maximization method, Eqs. (2,3) and Eqs. (15,16,23,24) (Sect. “Value of exhaustive or selective search in a large tree with rational* p*” of the Methods), and dots correspond to Bellman - Monte Carlo simulations (see Sect. “Bellman–Monte Carlo simulations” of the Methods; average over $10^4$ runs). The red lines in the two panels are identical. Errors bars are smaller than dots.

We have studied the value of playing trees as a function of *b*, *d* and *p* using the diffusion-maximization method in Eqs. (2,3) for $p=\frac{1}{2}$ and Eqs. (15,16) and (23,24) in the Methods for the rational values $p=\frac{n}{n+1}$ and $p=\frac{1}{n+1}$ with positive integer *n*. In all cases, the zero-average constrained is satisfied by setting $R_+=1$ and $R_- = -p/(1-p)$. The analytical predictions allow us to study very deep trees with, e.g., $d=20$ and $b=5$ at little numerical cost, where the number of nodes is larger than $2 \; 10^{13}$. In contrast, these digits are prohibitive for Bellman - Monte Carlo simulations. The value of playing a tree grows monotonically with both its depth and breadth (Fig. 4a), as a tree with a smaller depth or breadth is a sub-tree that can only have a value equal or smaller than the original tree. Asymptotically, the value grows with unit slope and runs parallel and below the diagonal line (dashed line), which constitutes the highest possible value of any tree, as no tree can have a value above it given our choice $R_+=1$. With larger *b*, the value runs closer to the diagonal. The value of the tree grows monotonically with the probability *p* of finding high expected reward nodes (Fig. 4b).

#### Selective allocation

Now we turn to the central problem of how to optimally sample an infinitely large tree with finite sampling capacity *C*. Assuming a tree having infinite number of levels and infinite branches per node allows us to consider any possible sampling allocation policy that is solely constrained by finite capacity. As such decision tree cannot be exhaustively sampled, we refer to the problem of allocating finite sampling capacity as ‘selective’ allocation. We restrict ourselves to a family of policies where the agent chooses the number of levels *d* that will be considered as well as the number of branches *b* per reached node that will be contemplated. Given finite capacity *C*, choosing a large *d* will imply having to choose a small *b*, thus allowing the agent to trade breadth for depth. To provide more flexibility to the allocation policy, we also allow that the agent chooses the probability $q_{d-l+1}$ of independently allocating a sample in each node in level $l \in \{1,\ldots ,d\}$ (note the reversed order, e.g., $q_1$ refers to the last level *d*). Under this stochastic allocation policy, a node receives a maximum of one sample or can receive none, and thus the allocation is an independent Bernoulli process with sampling probability $q_{d-l+1}$ in each node in level *l*. Note that here we have relaxed the hard capacity constraint to an average capacity constraint, which turns to be easier to deal with and leads to a smoother analysis. We have observed through numerical simulations that results do not qualitatively differ between hard and average capacity constraints.

In the following, we first compute the value of sampling a tree of depth *d* and branching factor *b* with per-level sampling probabilities $q=(q_1,\ldots ,q_d)$. The capacity constraint will be imposed afterward simply by constraining *d*, *b* and *q* to be such that on average the number of allocated samples equals capacity *C*. The algorithm is simply a generalization of the diffusion-maximization algorithm derived for exhaustive allocation in Eqs. (2,3), shown here for the case $p=\frac{1}{2}$ and generalized in Sect. “Value of exhaustive or selective search in a large tree with rational* p*” of the Methods to other rational probabilities.

In contrast to exhaustive allocation, when using selective allocation some nodes might not be sampled, as $q \le 1$, and thus they will remain having expected reward $R(s)=0$. As before, sampled nodes have values $R(s)=\pm 1$ with probability $\frac{1}{2}$. Therefore, the value $J_1$ of a depth-1 tree is in the set $\{-1,0,1\}$. To compute the expectation of $J_1$ we note that the action-value $Q_1$ of each branch (leaf) has values $\{-1,0,1\}$ with probabilities $P(Q_1=1)=\frac{1}{2} q_1$, $P(Q_1=0)=1-q_1$ and $P(Q_1=-1)=\frac{1}{2} q_1$, which follows from the facts that the node is sampled with probability $q_1$, that if it is sampled then its expected reward $R(s) = \pm 1$ with probability $\frac{1}{2}$, and that if it is not sampled then its expected reward is $R(s) = 0$. As *b* branches are available each with the same independent distribution of action-values, the value $J_1$ has probabilities $P(J_1=k)=P(Q_1 \le k)^{b}-P(Q_1 \le k-1)^{b}$, which results in $P(J_1=1) = 1- (1-\frac{q_1}{2})^b$, $P(J_1=0) =(1-\frac{q_1}{2})^b - (\frac{q_1}{2})^b$ and $P(J_1=-1) = (\frac{q_1}{2})^b$.

To compute $P(J_{d})$ recursively from $P(J_{d-1})$, we first relate $P(J_{d-1})$ with $P(Q_{d})$. Since the action-value can be written as $Q_{d}=R_d+J_{d-1}$, where $R_d$ is the reward in a node in level *d*, the diffusion step takes the form

4 $$\begin{aligned}&P(Q_{d}=d) = \frac{1}{2} q_d P(J_{d-1}=d-1)\nonumber \\&P(Q_{d}=d-1) = (1-q_d) P(J_{d-1}=d-1) + \frac{1}{2} q_d P(J_{d-1}=d-2)\nonumber \\&P(Q_{d}=d-2) = \frac{1}{2} q_d P(J_{d-1}=d-1) + (1-q_d) P(J_{d-1}=d-2) + \frac{1}{2} q_d P(J_{d-1}=d-3)\nonumber \\&\vdots \\&P(Q_{d}=2-d) = \frac{1}{2} q_d P(J_{d-1}=1-d) + (1-q_d) P(J_{d-1}=2-d) + \frac{1}{2} q_d P(J_{d-1}=3-d) \nonumber \\&P(Q_{d}=1-d) = \frac{1}{2} q_d P(J_{d-1}=2-d) + (1-q_d) P(J_{d-1}=1-d)\nonumber \\&P(Q_{d}=-d) = \frac{1}{2} q_d P(J_{d-1}=1-d)\;.\nonumber \end{aligned}$$

The diffusion step is followed by the maximization step

5 $$\begin{aligned} P(J_{d}=k) = P(Q_{d} \le k)^{b} - P(Q_{d} \le k-1)^{b}, \end{aligned}$$

for $k \in \{-d, -d+1,\ldots , d-1, d \}$. Iterating the diffusion and maximization steps in Eqs. (4,5) with initial conditions $P(J_1)$ described above allows us to compute $V_{d,b,q} = {\mathbb {E}}(J_{d})$, which is the value of playing a tree of depth *d*, branching factor *b* and per-level sampling probabilities *q*.

We now turn to the problem of optimizing *d*, *b* and *q* under the finite capacity constraint. In practice, we can consider a fixed, large *d* and optimize *b* and *q*, such that we effectively assume that the sampling probabilities are zero above some depth *d*. If *d* is large enough this assumption does not impose any restrictions, as the sampling probability can also be zero in levels shallower than the last considered level *d*. As the agent is limited by finite sampling capacity, both *b* and *q* are constrained by

6 $$\begin{aligned} C=\sum _{l=1}^d q_{d-l+1} \; b^{l} \;, \end{aligned}$$

which states that the average number of sampled nodes in the sub-tree must be equal to capacity *C*. The optimal *b* and *q* are found by

7 $$\begin{aligned} (b^*,q^*) = \mathop {{\mathrm{arg\,max}}}\limits _{b,q} V_{d,b,q} \;, \end{aligned}$$

subject to the capacity constraint, Eq. (6), and for large enough *d*. Optimal allocation policies are numerically found by using a gradient ascent algorithm (Sect. “Gradient ascent” of the Methods).

In addition to the optimal allocation policies in Eq. (7), that we call *heterogeneous*, we also consider a subfamily of selective allocations that we call *homogeneous*. In a homogeneous allocation policy, the sampling probability is one for all levels except, possibly, the last level, which is chosen to satisfy the finite capacity constraint. As shown below, homogeneous policies are close to optimal and are also simpler to study. In a homogeneous selective policy, as in exhaustive allocations, the only choice of the agent is the number of considered branches per reached node *b*. Then, effectively, upon choosing *b*, the agent samples *b* nodes in the first level, and from each of those the agent samples another *b* nodes in the second level, and so on until capacity is exhausted at some depth $d'\equiv d(b,C)$, that depends on *b* and *C*. Possibly, not all $b^{d'}$ resulting nodes in the last sampled level $d'$ can be fully sampled. Defining $C_r=C - \sum _{l=1}^{d'-1} q_{d'-l+1} b^{l}$ as the remaining number of samples available when reaching the last sampled level $d'$, then each of the $b^{d'}$ considered nodes is given a sample independently with probability $q_1' \equiv q_1(b,C)=C_{r}/b^{d'}$, such that on average total capacity equals *C*. More specifically, we focus on policies where *b* is free, $q_1'=C_{r}/b^{d'}$, and $q_2'=...=q_d'=1$ (note again reversed index), with $C_r > 0$. Within this family of allocation policies, the optimal policy is

8 $$\begin{aligned} b^* = \mathop {{\mathrm{arg\,max}}}\limits _b V_{d',b,q'} \;, \end{aligned}$$

where $V_{d,b,q}={\mathbb {E}}(J_{d})$ is found by using the diffusion-maximization method in Eqs. (4,5) and Eqs. (17,18,25,26) in Sect. “Value of exhaustive or selective search in a large tree with rational* p*” of the Methods.

### Optimal breadth-depth tradeoffs in allocating finite capacity

**Figure 5:**
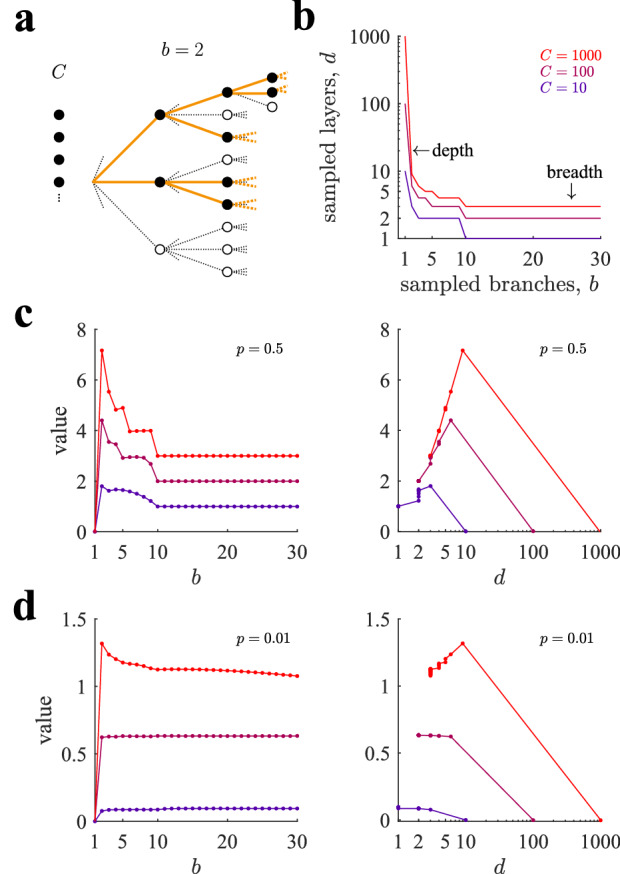
Optimal breadth-depth tradeoffs in sampling decision trees with finite capacity. (**a**) An agent chooses the number of branches that will be sampled, *b*, per reached node from the root node and continues to sample the tree until capacity is exhausted (*homogeneous* selective allocation). The last nodes are sampled stochastically, so that on average the number of samples equals capacity *C*. In the example the number of sampled branches is $b=2$. (**b**) At fixed capacity, there is a tradeoff between the number of sampled branches and the number of sampled levels. Three values of *C* have been chosen ($C=10,100,1000$), representing low, medium and high search capacity. For the same number of sampled branches, the number of sampled levels increase with *C*. The number of sampled levels includes the last level, which might only be partially sampled. Transitions between plateaus occur when the last level is filled up completely with samples. (**c**) Left panel: Value of playing the tree by choosing to sample *b* branches per reached node with three different values of capacity for $p=\frac{1}{2}$. Note that for each line, selecting *b* determines the depth of the played tree *d* (see panel (b)) due to the finite capacity constraint. The optimal value is attained when the number of sampled branches is $b=2$. Right panel: same data as in the right panel are re-plotted as a function of the depth *d* of the considered sub-tree. The second longest depth allowed given finite capacity is the optimal allocation to play the tree, which corresponds to $b=2$ in the left panel. The curve shows some vertical jumps because the tree value changes as a function of *b* even though it does not change *d*. (**d**) Same as in panel (c) for $p=0.01$. While at high capacity sampling the tree with a low number of sampled branches remains optimal, at lower capacities it is best to play the tree by favoring breadth over depth. In all panels, points correspond to simulations (average over $10^6$ runs) and solid lines correspond to theoretical predictions by Eqs. (4–6) and Eqs. (6,17,18,25,26) (Sect. “Value of exhaustive or selective search in a large tree with rational* p*” of the Methods) for the homogeneous allocation case.

We now describe how optimal selective allocations depend on sampling capacity *C* and on the richness of the environment as measured by *p*. We start by homogeneous policies, which will be shown in the next section to be very close to optimal when compared to heterogeneous policies. Selective homogeneous allocations maximize the value of sampling selectively an infinitely broad and deep tree by optimizing the number of sampled branches *b* (Eqs. 4,5,8). As capacity is constrained and the sampling probability is one except possibly for the last level, choosing a large *b* implies reaching shallowly in the tree (Fig. 5b). Thus optimal BD tradeoffs are reflected in the optimal number of considered branches. We find that the optimal number of branches is $b^*=2$ for a rich environment ($p=\frac{1}{2}$) regardless of capacity (Fig. 5c, left panel). Interestingly, we observe that choosing $b=1$ or $b=3$, which are the neighbor policies to the optimal $b^*=2$, leads to a large reduction of performance, indicating that the benefit from correctly choosing the optimum is high. The optimal $b^*=2$ favors exploring trees as deep as possible while keeping the possibility of choosing between two branches at each level. Indeed, the deepest possible policy resulting from the policy $b=1$ is highly suboptimal (leftmost point in the left panel, and rightmost points in the right panel), as the expected cumulative reward equals zero due to lack of freedom to select the best path.

For a poor environment (Fig. 5d; $p=0.01$), the optimal number of sampled branches is also $b^*=2$ when capacity is large (peak of red line), but as capacity decreases, $b^*$ increases. Thus, the optimal policy approaches pure breadth at low capacity, which entails exhausting all sampling resources in just the first level. We observe that in this case the dependence of the value of playing the tree with *b* is very shallow when capacity is small (blue line), and therefore the actual optimal $b^*$ is quite loose.

**Figure 6:**
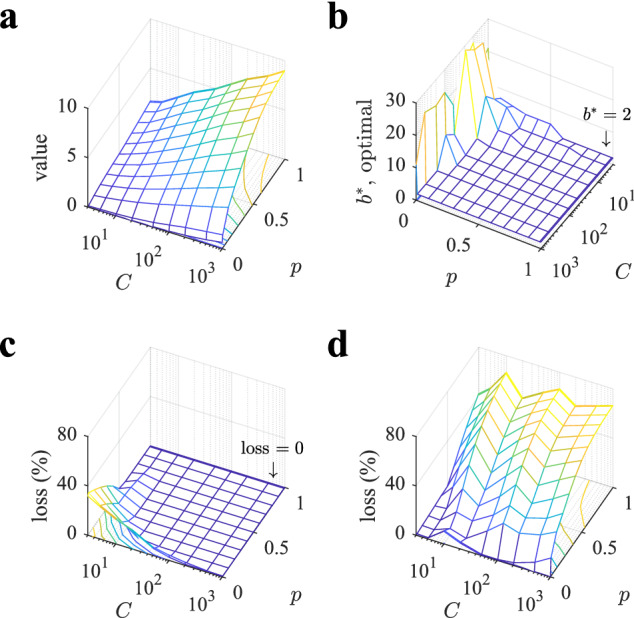
Depth dominates over breadth in large regions of the parameter space. (**a**) Value of playing optimally a tree as a function of capacity *C* and probability *p*. (**b**) Optimal number of sampled branches $b^*$ as a function of *C* and *p* (note that *C* and *p* axes have been rotated for a better data visualization). The large plateau corresponds to the optimal number of sampled branches $b^*=2$. (**c,d**) Loss incurred in playing the tree always with $b=2$ (c), corresponding to depth sampling, or with $b=20$ (d), corresponding to breadth sampling. The large plateau in panel (c) corresponds to loss equal to zero. Losses are defined as $100 (V_{opt}-V)/V_{opt}$, where $V_{opt}$ is the optimal value (from panel a) and *V* is the value of sampling the tree with the corresponding heuristic. Bellman - Monte Carlo simulation results are averaged over $3 \; 10^6$ repetitions.

The results for the two environments described above suggest that depth is always favored when capacity is large enough or whenever the environment is rich, while breadth is only favored at low capacities and for poor environments. Further, while optimal breadth policies can be quite loose in that choosing the exact value of *b* is not very important to maximize value, optimal depth policies are very sensitive to the precise value of the chosen value *b*, always very close to $b=2$, such that variations of it cause large losses in performance. Exploration of a large parameter space confirms the generality of the above results (Fig. 6). In particular, the optimal number of sampled branches is $b^*=2$ for a very large region of the parameters space (Fig. 6b), while an optimal number of branches larger than 2 mostly occurs exclusively when *p* is small ($p < 0.1$) or capacity is small ($C<10$). If the agent used a depth heuristic consisting in always sampling 2 branches, then the loss incurred compared to the optimal *b* would be around $40\%$ at the most, but the region where there are significant deviations in performance concentrates at both low *C* and *p* values (Fig. 6c). Indeed, for a very large region of parameter space the loss is zero because almost everywhere the optimal number of sampled branches equals 2 or because the value of playing the tree is not very sensitive to *b*. In contrast, using a breadth heuristic where the agent always uses $b=20$ is almost everywhere a very poor policy, as losses can reach close to or above $40\%$ in large regions of the parameter space (Fig. 6d). Therefore, as an optimal strategy, depth dominates over breadth in larger portions of parameter space, and as a heuristic, depth generalizes much better than breadth.

Although the optimal policy is quite nuanced as a function of the parameters, a general intuition can be provided about why depth tends to dominate over breadth: exploring a tree allows agents to find paths with cumulative reward bounded by the length of the path; thus, exploring more deeply leads to knowledge about potentially large rewards excesses as compared to exploring less deeply and following afterward a default policy. Although this effect seems to be the dominant one, being able to compare among many short courses of action becomes optimal in poor environments when capacity is small, as it allows securing at least a good enough cumulative reward.

### Exploring further into the future is a slightly better policy

**Figure 7:**
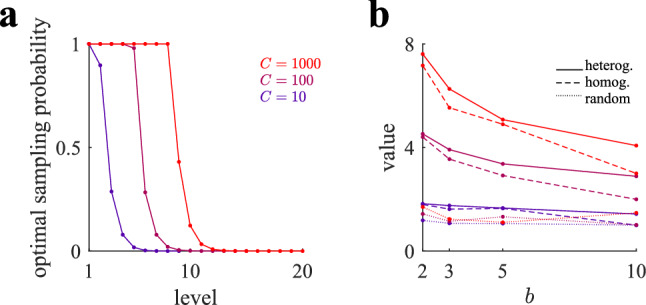
Optimal heterogeneous policies spread samples into the future more deeply than homogeneous policies. (**a**) Optimal sampling probabilities *q* per level for three capacities and for $b=2$. While for optimal homogeneous policies sampling probabilities equal one except, possibly, for the last level, optimal heterogeneous policies assign non-zero sampling probabilities to deeper levels. (**b**) Value of playing the tree with heterogeneous (full lines), homogeneous (dashed) and random (dotted) policies as a function of the number of considered branches *b* for three capacities (color code as in previous panel). The optimal value is attained when $b=2$ for all cases. Note that optimal values for homogeneous policies are below but very close to the optimal values of heterogeneous policies. For heterogeneous and random policies, we limit the number of considered levels somehow arbitrarily to $d = 2 \lfloor ln(C)/ln(b) \rfloor + 3$, where $\lfloor x \rfloor$ is the floor function, which allows in a simple way agents to spread samples, if optimal, well beyond the sampled levels by homogeneous policies. Random policies allocate samples with the same probability to every node of the tree of depth *d* and also satisfy the finite capacity constraint, Eq. (6). Optimal policies and values for heterogeneous and for homogeneous selective allocations are computed using Eqs. (4,5,7) and Eqs. (4,5,8) for $p=\frac{1}{2}$, respectively, inside a gradient ascent (see Sect. “Gradient ascent” of the Methods). For different *p* results are similar.

One important question is how much can be gained by giving to the agent a larger degree of flexibility in allocating samples over the levels. In heterogeneous selective policies, the agent is free to choose the number of branches to be considered as well as the sampling probabilities for each of the levels (Eqs. 4,5,7). Therefore, in contrast to homogeneous selective policies, the agent can decide not to allocate samples to the first levels and reserve them for deeper levels. Our analysis, however, shows that it is not the best allocation policy, as optimal heterogeneous policies exhaustively sample the first levels, as homogeneous policies do (Fig. 7a). One important difference is that optimal heterogeneous policies explore further into the future than homogeneous policies. This is accomplished by using sampling probabilities decaying to zero in the last few sampled levels. This is in contrast to homogeneous policies, where only the last level is given, possibly, a sampling probability smaller than one. Thus, exploring slightly further into the future provides a surplus value of playing the tree (Fig. 7b, full lines), but it is only marginally better than the one obtained from homogeneous policies (dashed lines), which are much simpler to implement due to their fixed sampling probability structure. As in the case of homogeneous policies, heterogeneous policies attain their optimal value when the number of considered branches is 2, thus favoring depth over breadth search. Finally, we tested random policies where samples are allocated with the same probability to the nodes of the first levels of the tree until capacity is exhausted (dotted lines), and found that they are much worse than the optimal policies.

### Even deeper allocation policies are generally best

The previous results show that allocations that deeply sample into the tree are favored under a large variety of circumstances. A limitation of the allocations that we have used so far is that even a branching factor of $b=2$ can made the sampled tree very wide at deep levels. Therefore, we wondered whether even deeper allocations strategies would be favored by allowing that two different branches can be used: a first one $b_1$ applied over the first $d_1$ levels, after which a branching factor of $b_2$ would be used until capacity is exhausted. We restrict our analysis to homogeneous allocation policies, as heterogeneous ones generally provide a marginal improvement respect to the the first ones, as shown in the previous section. We characterize the optimal $b_1$, $b_2$ and $d_1$ using Eqs. (4,9,12) in Sect. “Two-*b* allocation” of the Methods as a function of the sampling capacity *C* and the probability *p* defining the richness of the environment.

**Figure 8:**
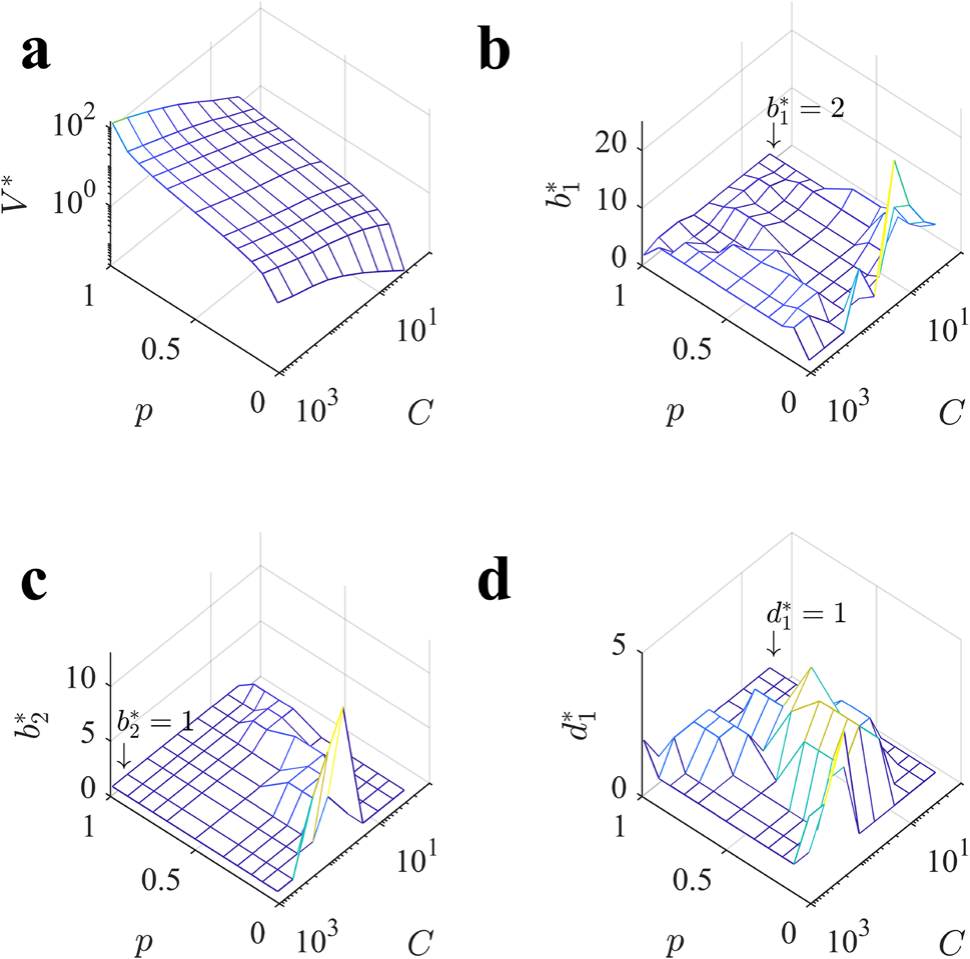
Optimal two-*b* policies favor deep allocations in most of the parameter space. (**a**) Value of playing optimally a tree $V^*$ as a function of capacity *C* and probability *p* with the optimal set of parameters $(b_1^*,b_2^*,d_1^*)$. (**b**) Optimal first branching factor $b_1^*$ as a function of *C* and *p*. For most of the parameter space, the optimal $b_1$ is close or equal to 2. (**c**) Optimal second branching factor $b_2^*$. The large plateau corresponds to $b_2^*=1$. Larger values of $b_2$ are optimal only in very poor environments. (**d**) Optimal switching depth $d_1^*$. When large resources are available we find $d_1^*=1$, namely, it is optimal to switch from $b_1^*$ to $b_2^*=1$ after the first level and explore few long not-branching paths. Contrariwise, at low and intermediate values of *C*, most of the samples should be allocated using the first branching factor $b_1^*$ and little role will be played by $b_2^*$. In all panels, surfaces correspond to the theoretical predictions by Eqs. (12,19,20) and Eqs. (12,27,28) (Sect. “Value of exhaustive or selective search in a large tree with rational* p*” of the Methods).

Results for the two-*b* policies confirm our previous results, but also reveal a rich set of behaviors that depart from them: we find again that it is almost always optimal to allocate samples with $b_1\sim 2$ (Fig. 8b), with the exception of poor environments with small capacity. More interestingly, when the agent is allowed to consider a different branching factor for deeper levels, it is optimal to sample even fewer nodes per level, as $b_2\sim 1$ is optimal in most of the parameter space (Fig. 8c). This policy corresponds to sampling very few but deep paths with little branching. Deviations from this behavior are found again in poor environments, where larger values of $b_2$ become optimal.

In this family of allocation policies, a relevant role is played by the optimal depth $d_1^*$ of switching from one branching factor to the other (Fig. 8d). In poor environments and with low capacity, $b_1^*$ is larger than the capacity itself, namely, there is little benefit from a switching strategy, and the same values would have been obtained by using any arbitrary $b_2$. The optimal $d_1^*$ then coincides with the depth of the tree and makes $b_2$ irrelevant. This result is consistent with what we previously discussed in the single-*b* allocations, where we found that at low capacity and poor environments breadth dominates over depth. With higher capacity, however, it is best to switch soon towards the second branching factor $b_2$. Taken together, the optimal strategies shown for $b_1, b_2$ and $d_1$ highlight even more the optimality of deep allocations observed so far, with few ($b_1^*\sim 2$) not branching ($b_2^*\sim 1$ and $d_1^*\sim 1$) paths to be sampled as the preferred allocation.

**Figure 9:**
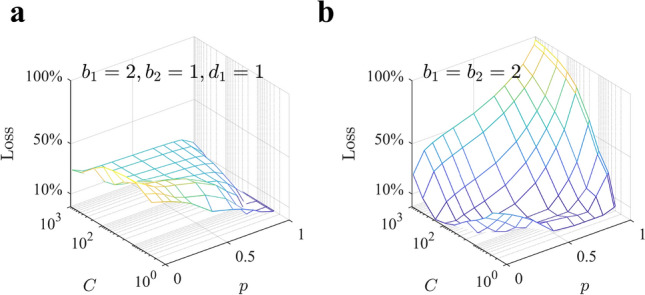
Loss incurred in playing the tree always with a specific heuristics instead of the optimal two-*b* policy. (**a**) Relatively restrained loss occurs using as a heuristics the deep allocation policy $(b_1,b_2,d_1)=(2,1,1)$. (**b**) Optimal two-*b* policies clearly outperform the generally optimal single-*b* policy with parameters $b=b_1=b_2=2$. Loss is defined as $100(V_{opt}-V)/V_{opt}$, where $V_{opt}$ is the optimal value from Fig. 8a and *V* is the value corresponding to the specific heuristics. Results are obtained with the theoretical predictions by Eqs. (12,19,20) and Eqs. (12,27,28) (Sect. “Value of exhaustive or selective search in a large tree with rational* p*” of the Methods).

We tested the performance of this ‘very-deep-heuristics’ by allocating samples in two long paths ($b_1=2$, $d_1=1$ and $b_2=1$) in all the parameter space (Fig. 9a) and compare its value with that of the optimal policy for each parameter value in Fig. 8a. The loss the agent faces is relatively low, standing around $20\%$ in most of the parameter space. As we would expect, the biggest losses are found at very poor environments and low capacity, where breadth dominates over depth.

Another relevant question is how much value it is gained by allocating samples with two different branching factors instead of a single one. The loss that the agent incurs in by sampling always with $b=b_1=b_2=2$ is large with respect to the optimal set of variables in two-*b* homogeneous policies, and increases with both *C* and *p* up to relative losses of $100\%$, the maximum possible (Fig. 9b). We conclude that in a large region of the parameter space, it is disproportionately better to allocate samples according to very-deep two-*b* allocations. Consistent with our initial intuition, with a single *b* to be chosen, the agent cannot reach very deep into the tree even for $b=2$ due to the exponential grow of sampled nodes with tree depth; however, with a two-*b* policy this is possible, which allows the agent to observe deeper paths, thus increasing the expected cumulative rewards discovered along them in a large portion of the parameter space.

### Deep allocation is optimal for deep enough trees

**Figure 10:**
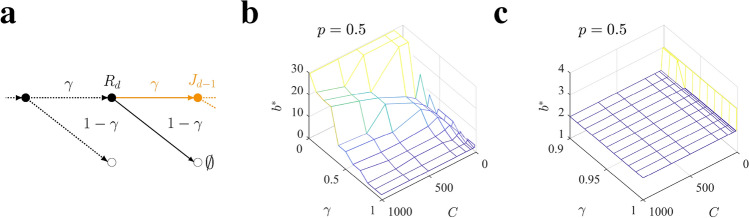
Depth dominates in a discounted setting in deep enough trees. (**a**) Scheme of the discounted algorithm. At level *d*, the agent gains the immediate reward $R_d$ and only with probability $\gamma$ they are able to collect the accumulated reward $J_{d-1}$ in the future path (orange). On the contrary, with probability $1-\gamma$ they end up in the null absorbing state with zero contribution (black, solid arrow). See Sect. “Discounted setting” of the Methods for the details and the description of the analytical solution. (**b,c**) Optimal branching factor $b^*$ in the discounted setting for selective homogeneous allocation policies as a function of the temporal discount factor $\gamma$ and the available resources *C* in an environment with $p=0.5$. The temporal discount strongly affects the optimal policy, reducing the agent horizon to a single or two levels and accordingly favoring wide allocations (b). Nevertheless, deep allocations continue to be optimal for the most relevant range $0.9\le \gamma \le 1$ (c), where future levels of the tree can be sampled. Results are obtained with the theoretical predictions by Eqs. (5,13,14) in Sect. “Discounted setting”and Eqs. (14,21,22) and Eqs. (14,29,30) in Sect. “Value of exhaustive or selective search in a large tree with rational* p*” of the Methods.

In the previous examples, a bias towards preferring depth over breadth allocations originates due to the fact that rewards are accumulated over the chosen path. To reduce this bias, we introduced a temporal discount, such that rewards at *l* levels in the future are exponentially less relevant that the same rewards close in the future by a factor $\gamma ^l$. We restrict our analysis to homogeneous allocation policies with a single branching factor, as they have proved to favor deep allocations in most of the conditions, and we show here how our former results are robust against the introduction of the temporal discount for standard values. We characterize the optimal branching factor $b^*$ using Eqs. (13, 5, 14) described in Sect. “Discounted setting” of the Methods (see Fig. 10a for a scheme of the discounted algorithm) as a function of the sampling capacity *C* and discount $\gamma$.

We find that the discount factor $\gamma$ has a strong effect on the optimal policy. For $\gamma > 0.9$ the optimal branching factor is close to 2 for the whole range of capacities tested (Fig. 10b,c), and therefore deep allocation is preferred in this range. However, for smaller values of $\gamma$, the optimal branching factor become much larger so that breadth starts to dominate (Fig. 10b). These results qualitatively hold for all values of the environmental parameter *p*. It is important to note that introducing a discount factor reduces the depth of the tree to $1/(1-\gamma )$. For this reason, a temporal discount of, e.g., $\gamma \sim 0.5$ makes the effective depth of the tree $1/(1-\gamma ) = 2$, such that effectively samples allocated beyond level 2 are discarded, a fact that explains why breadth dominates in the low $\gamma$ regime. In the same way, when $\gamma > 0.9$ the effective depth of the tree is larger than 5, and therefore more distant rewards can have a sizable effect on the tree value when sampled. Interestingly, the range $\gamma > 0.9$ where depth dominates coincides with the standard range considered in the literature^14,32^. In summary, in deep enough trees ($\gamma$ close to one), deep allocation dominates over breadth for all values of capacity and for all environments tested.

## Discussion

Agents with limited resources face breadth-depth tradeoffs when looking for the best course of action in deep and wide decision trees. To gain information about the best course, an agent might allocate resources to sample many actions per level at the cost of not exploring the tree deeply, or allocate resources to sample deeply the tree at the risk of missing relevant actions. We have found that deep imagination is favored over breadth in a broad range of conditions, with very little balance between the two: it is almost always optimal to sample just one or two actions per depth level such that the tree is explored as deeply as possible while sacrificing wide exploration. In addition, using depth as a heuristic for all cases incurs much smaller errors than assuming a breadth heuristic. We have provided analytical expressions for this problem, which allows us to study the optimal allocations in very large decision trees.

During planning, we very often picture the course of action as an imaginary episode, from taking the plane to visiting the first museum, in a process that has been called imagination-based planning, model-based planning, mental simulations or emulation, each term carrying somehow different meanings^24,27,33–37^. Imagination strongly affects choices through the availability of the imagined content^25^, and it is used when the value of the options are unknown and thus preferences need to be built on the fly^24^. However, imagination-based planning is slow and there is no evidence that can run in parallel^38,39^, implying that as an algorithm for exploring deep and wide decision trees it might not be efficient. Indeed, very few courses of action ($\sim 5-10$) are considered in our ‘minds’ before a decision is made^40–45^, and in some cases the imagined episodes can be characteristically long, like when playing chess^22^, although their depth can be adapted to the current constraints and time pressure^46^. As an alternative to its apparent clumsiness, *deep imagination* –the sampling of few long sequences of states and actions– might have evolved as the favored solution to breadth-depth tradeoffs in model-based planning under limited resources against policies that sample many short sequences. With this new terminology, we intend to turn the spotlight on the process of investing resources according to the internal knowledge and before any feedback is collected. Our result that depth allocations dominate over a broad range of capacity and environmental parameters provides a theoretical foundation for the optimality of deep imagination in human model-based planning. Recent deep-learning work has studied through numerical simulations how agents can benefit from imagining future steps by using models of the environment^47–50^, and thus our results might help to clarify and stress the importance of deep tree sampling through mental simulations of state transitions.

Deep imagination resembles depth-first tree search algorithms in that they both favor deep over broad exploration^8,51^. However, depth-first search starts by sampling deeply until a terminal state is found, but actually reaching a leaf node in very deep trees can be unpractical^15^ and even the notion of leaf node might not be well-defined, as in continuing tasks^14^. In very deep decision trees such strategy would imply the sampling of a single course of action until exhaustion of resources, which is a highly suboptimal strategy, as we have shown (see Fig. 5 with $b=1$). Another family of search algorithms, called breadth-first search^8^, and other approaches that give finite sampling probability to every action at each visited nodes, such as Monte Carlo tree search^15^ or $\epsilon$-greedy reinforcement learning methods^14^, can poorly scale when the branching factor of the tree is very large, and thus they are unpractical approaches for BD dilemmas. In contrast, deep imagination samples one or two actions per visited node until resources are exhausted, which allows selecting the best among a large number of long paths, and at the same time constitutes an algorithm that is simple to implement and generalizes well. Due to finite capacity, any algorithm can only sample a large decision tree up to some finite depth, which leaves open the question of how the agent should act afterward. Following the approach of plan-until-habit strategies^46,52^, we have assumed that agents can follow a random, or default, strategy after the last sampled level of the tree, such that different allocation policies with different sampled depth and branching factors could be compared on an equal footing.

As mentioned before, previous research in tree search optimization has focused on problems where rewards are available at the end leaves, but intermediate states do not confer any reward per se^15,30,31,46,53^. By collecting rewards only at the leaf nodes, this framework requires a finite horizon and is not suitable to model many real life decisions where the overall value of a path has contributions coming from different levels. In these cases, an accumulated reward framework may be preferred^18,52,54^. Our work aligns with this hypothesis by efficiently computing the expectations of the optimal accumulated rewards using different allocation strategies. By construction, this assumption suffers a bias towards a deep exploration of the tree. However, we have shown how, even when reducing this bias with the introduction of a discount factor, deep allocations may still be favored in most of the conditions.

One assumption of our model is that rewards are distributed independently and identically over the tree. However, in most interesting problems the rewards coming from neighboring leaf nodes are correlated^20,55–57^. Indeed, correlations between nodes within levels would be a realistic feature that could be a priori added in our modeling approach. Although further research would be required, we think, however, that the dominance of deep allocations might continue to hold with correlated rewards, for the following reason: correlated rewards within a level will favor learning about them by sampling very little per level, and therefore deep allocation will be favored even more.

Another important assumption in our work is the one-shot nature of the sample allocation. Many important decisions have delayed feedback, like allocating funding budget to vaccine companies, choosing college, or planning a round of interviews for a faculty position, and thus they are well modeled as one-shot finite-resource allocations^40,42,43^. However, other decisions involve quicker feedback and then the allocation of resources could be adapted on the fly. Although our results are yet to be extended to sequential problems where at every step a compound action is to be made, we conjecture that such extension will not substantially change the close-to-optimality of deep sampling, although a bias towards more breadth is expected^4^. Further, pre-computing allocation strategies at design-time and using them afterward might lift up the burden of performing heavy online computations that would require complex tree expansion in large state spaces. Thus, by hard-wiring these strategies much of the overload caused by meta-reasoning^1–3^ could be alleviated, allowing agents to use their finite resources for the tasks that change on a faster time scale. Finally, it is important to note that, in contrast to many experimental frameworks on binary choices or very low number of options^58–61^ and games^22,62^ where the number of actions is highly constrained by design, realistic decisions face too many immediate options to be all considered^40–42,45^, and thus a first decision that cannot be deferred is how many of those to focus on in the first place^4,10,61,63^. All in all, the optimal BD tradeoffs that we have characterized here might play an important role even in cases that substantially depart from our modeling assumptions.

In summary, we have provided a theoretical foundation for deep imagination as a close to optimal policy for allocating finite resources in wide and large decision trees. Many of the features of the optimal allocations that we have described here can be tested by controlling parametrically the available capacity of agents and the properties of the environment^10^ by using similar experimental paradigms to those recently developed^11,53^.

## Methods

### Model details

Here we provide a more formal description of the decision tree model sampled with finite capacity. We consider a Markov Decision Process (MDP) that operates in two consecutive phases having different actions (Fig. 1b). The first phase is a learning or exploration phase, while the second one is an exploitation phase. In both phases, the underlying structure is a directed rooted tree ${\mathcal {G}}=({\mathcal {V}},{\mathcal {E}})$ with *d* levels and homogeneous branching factor, or out-degree, *b*. Thus, each parent node has exactly *b* children so that there are $b^{k}$ nodes at level $k \in \{0, 1,\ldots ,d\}$. Both *b* and *d* can be made to grow to generate an infinitely large tree. Vertices in ${\mathcal {V}}$ correspond to nodes in the tree, with a total of $|{\mathcal {V}}|=(b^{d+1}-1)/(b-1)$ of them, and edges ${\mathcal {E}}$ are links between parents and their *b* children nodes. In the first phase, an action consists of sampling in one shot a subset of $C \le |{\mathcal {V}}|-1$ nodes in ${\mathcal {G}}$ excluding the root node, denoted ${\mathcal {V}}_{\text {sampled}} \subset {\mathcal {V}}$, which results in observing the associated random variables $X_s$ for each $s \in {\mathcal {V}}_{\text {sampled}}$. The random variables are independently and identically distributed as a binary variable with success probability *p*, and their values are hidden to the observer before sampling. Based on the outcomes of the sampled nodes, the agent can update their belief about the expected rewards that would result from actually visiting them, *R*(*s*) for all $s \in {\mathcal {V}}_{\text {sampled}}$, while the expected reward *R*(*s*) resulting from visiting unsampled nodes $s \in {\mathcal {V}}_{\text {unsampled}}$ remains unchanged. In the second phase, the agent lies on a standard MDP over ${\mathcal {G}}$. Here, edges correspond to actions, $a \in {\mathcal {E}}$, each leading to a deterministic transition along the edge between the parent and one children. The expected reward resulting from visiting state $s \in {\mathcal {V}}$ in the tree are the *R*(*s*)-s updated (or not) in the first phase. The goal of the agent is to optimize the allocation of samples such that the expected cumulative reward amongst the best possible path is maximized. Next we describe the above in further detail and provide a rationale for our modeling choices.

In the learning phase, we assume that the agent has a finite search capacity, modeled as a finite number of samples $C \le |{\mathcal {V}}|-1$ that can be allocated over the tree (Fig. 1b, orange panel). The most interesting scenario corresponds to $C \ll |{\mathcal {V}}|$, when the agent can only sample a small fraction of the nodes in a large decision tree. Thus, the agent’s action set equals all possible allocations of the *C* samples over the graph ${\mathcal {G}}$ excluding the root node. Formally, every node $s \in {\mathcal {V}}$ has an associated binary variable $n_s \in \{0,1\}$, indicating whether the node has been sampled, $n_s=1$, or not, $n_s=0$. Note that we assume that nodes can be sampled at most once, and that the finite capacity constraint imposes $\sum _s n_s = C$. Then, the action set can be expressed as ${\mathcal {A}}=\{(n_1,n_2,\ldots ,n_{|{\mathcal {V}}|-1}): \sum _s n_s = C , n_s \in \{0,1\} \}$. The nodes with $n_s=1$ define the subset of sampled nodes ${\mathcal {V}}_{\text {sampled}} \subset {\mathcal {V}}$. Finite sampling capacity models cognitive and time limitations of the agent, which impedes that a full exhaustive search over all the nodes be possible.

The result of sampling a node *s* is to gain information about the expected reward *R*(*s*) that would result from actually visiting the node, which will used in the exploitation phase to optimize the course of action. We assume that, before sampling starts, the expected reward of any state *s* is $R(s)=0$. With this choice, if the agent chose any path from the root to the leaves and navigated thought it without having sampled any of the nodes before, the expected cumulative reward associated to such course of action would be zero.

When the agent chooses an allocation action $a \in {\mathcal {A}}$, the graph is partitioned into the sampled and unsampled nodes, ${\mathcal {V}}_{\text {sampled}}=\{s : n_s = 1\}$ and ${\mathcal {V}}_{\text {unsampled}}=\{s : n_s = 0\}$ (excluding the root node), respectively. The expected reward of an unsampled node, $n_s=0$, is not updated and thus it remains $R(s)=0$. For a sampled node, $n_s=1$, the belief about its expected reward is updated as follows: we assume that the outcome of sampling the node *s* is to update the expected reward *R*(*s*) from expected reward 0 to expected reward $R_+$ with probability *p* and to expected reward $R_-$ with probability $1-p$, independently for each sampled node (see Fig. 1b, blue and red dots). Thus, $P(R(s)=R_+|n_s=1)=p$ and $P(R(s)=R_-|n_s=1)=1-p$ for a sampled node, and $P(R(s)=0|n_s=0)=1$ for an unsampled node. We enforce the condition that the average over updated expected rewards equals zero, that is, $p R_+ + (1-p) R_-=0$, such that sampling a node does not result in net reward or loss (‘zero-average constraint’), which can be satisfied by taking $R_+=1$ without loss of generality and then using $R_- = -\frac{p}{1-p}$. This constraint is a form of the law of total expectation. The probability of a high reward *p* in a sampled node measures the overall richness of the environment.

Once the expected rewards have been updated, the optimal path (Fig. 1b, blue path) is computed, which corresponds to the one that has the highest expected cumulative reward based on the observations from the samples. Specifically, in the exploitation phase the decision problem forms a standard MDP ${\mathcal {M}}=({\mathcal {V}}, {\mathcal {E}}, {\mathcal {R}}, {\mathcal {T}})$, where states corresponds to nodes in the graph, $s \in {\mathcal {V}}$, actions correspond to edges of the graph, $a \in {\mathcal {E}}$, the learned rewards *R*(*s*) correspond to the actual expected rewards that result from visiting state *s*, and the transition function $T: (s,a) \rightarrow s'$ between states after an action is made is deterministic along the selected edge. The agent starts in the root node of ${\mathcal {G}}$, corresponding to the zero-*th* level, and takes action $a_1 \in \{1,\ldots ,b\}$, which results in a deterministic transition to the $a_1-th$ children node *s* in the first level and the acquisition of a reward with expected value *R*(*s*). Recursively, from node *s* at level *k*, the agent can choose a new action $a_{k} \in \{1,\ldots ,b\}$ resulting in a transition to its $a_k-th$ children node *s* in level $k+1$ and the acquisition of a reward with average *R*(*s*). At the $d-th$ level, there are not available actions and thus leaves correspond to terminal states. Given the learned expected rewards *R*(*s*), the optimal course of action is found by using backward induction^14^. As we will see, the optimal set of sampled nodes forms a much smaller tree than the original one due to the finite sampling capacity, and then backward induction over the reduced tree becomes tractable.

The overall goal of the agent is to determine the best policy to allocate *C* samples in order to maximize the expected cumulative reward of the optimal path.

### Bellman–Monte Carlo simulations

The exact values of playing tree for a subset of rational values of *p* are computed using the diffusion-maximization algorithm. For probabilities of positive rewards *p* not in that set, we can estimate the value by Bellman - Monte Carlo simulations. We first sample each node in the tree (except the root node) to determine the reward associated with it, *R*(*s*), which is $R(s)=R_+$ with probability *p* and $R(s)=R_-$ with probability $1-p$. We take $R_+=1$ and $R_- = -p/(1-p)$ to satisfy the zero-average constraint. Based on the learned *R*(*s*)-s, we compute the value of the tree by using backward induction from the last nodes until reaching the root node. Specifically, the leaf nodes have value $V(s)=R(s)$. Recursively, going backward, the value of a node *s* at depth *m* is computed from the values of its children nodes $s' \in \text {ch}(s)$ at depth $m+1$ as $V(s) = \max _{s' \in \text {ch}(s)} (R(s') + V(s') )$. The value of playing the tree with the specific realization of the *R*(*s*)-s is the value of the root node computed that way. The value of playing the tree is the average value over a large number of realizations of the *R*(*s*)-s, as indicated in the corresponding figures.

### Gradient ascent

For each *b* we optimize *q* in Eq. (7) under the capacity constraint, Eq. (6), by a gradient ascent method. The unconstrained gradient of the value $V_{b,d,q}$ is numerically computed for an initial *q* using a discretization step size $\Delta q_k=10^{-7}$, $k \in \{1,\ldots ,d \}$. The unconstrained gradient is then projected onto the capacity constraint plane defined by Eq. (6). Then, the projected gradient multiplied by a learning rate $\eta =10^{-3}$ is added to the original *q*, from where a new *q* is proposed. If the resulting *q* has a component $q_k$ that does not satisfy the constraint $0 \le q_k \le 1$, then $q_k$ is moved to either 0 or 1, whichever is closer. This movement can make *q* in turn to be outside the capacity constraint plane, so a new projection onto the constraint plain is performed. The projections and movements are repeated until *q* satisfies both constraints, leading to a new valid *q*. From the new *q*, an unconstrained gradient is computed again, and the procedure continues up to a maximum of $10^6$ iterations or when the improvement in the value $V_{b,d,q}$ is less than a tolerance of $10^{-9}$. To avoid numerical instabilities for very deep trees ($d>50$), the probabilities $P(J_d)$ are normalized to sum one at every iteration. One order of magnitude differences in the ranges of step sizes, learning rates and tolerances, and all tested initial conditions for *q* give almost identical results to those reported in the main text. Numerical analysis suggests that the value $V_{b,d,q}$ is a concave function of the *q* for fixed values of *b* and *d*, which could explain why the gradient ascent algorithm finds a single optimum under the linear capacity constraint in Eq. (6) regardless of initial conditions tested. We have been able to analytically confirm concavity of the value for the case $d=2$. We can also show the intuitive result that the value $V_{b,d,q}$ is a monotonically increasing function of the parameters *q*.

### Two-*b* allocation

We enrich the policy space of selective policies by letting the agent allocate samples in the first $d_1\ge 1$ levels with branching factor $b_1$ and with branching factor $b_2$ in the following $d_2$ until the resources are exhausted. We call this family of policies *two*-*b* allocation. This enlarged policy space incorporates the previously described allocations with single branching factor *b* as the particular case with $d_2=0$. To compute the value of playing a tree using this policy, we make use of a generalized version of the diffusion-maximization algorithm described in Eqs. (4,5) for selective allocations by introducing two different branching factors. We show here the case $p=\frac{1}{2}$ and leave to Sect. “Value of exhaustive or selective search in a large tree with rational* p*” of the Methods the generalization to rational values of probability. As before, we compute the expectation of the $J_1$ of a depth-1 tree from the action-value $Q_1$ of each leaf node, with probabilities $P(Q_1=1)=\frac{1}{2}q_1$, $P(Q_1=0)=1-q_1$ and $P(Q_1=-1)=\frac{1}{2}q_1$. At the bottom of the tree $b_2$ branches are available with the same independent distribution of $Q_1$, and therefore the value $J_1$ has probabilities $P(J_{1}=k) = P(Q_{1} \le k)^{b_2} - P(Q_{1} \le k-1)^{b_2}$ where $k\in \{-1,0,1\}$. $P(J_d)$ can be then computed recursively from $P(J_{d-1})$ by first relating $P(J_{d-1})$ with $P(Q_d)$ in the diffusion step as in Eqs. (4). The diffusion step is followed by the maximization step, taking here the form

9 $$\begin{aligned} P(J_{d}=k) = P(Q_{d} \le k)^{b_j} - P(Q_{d} \le k-1)^{b_j}\;, \end{aligned}$$

for $k \in \{-d, -d+1,\ldots , d-1, d \}$, where $b_j=b_2$ if $d\le d_2$ or $b_j=b_1$ if $d>d_2$, noticing that the algorithm runs backward by first facing the last $d_2$ levels sampled with branching $b_2$. By iterating Eqs. (4,9) we can compute the value of playing a tree of depth $d=d_1+d_2$ as $V_{d_1,d_2,b_1,b_2, q}={\mathbb {E}}(J_d)$.

We now turn to the problem of optimizing the free parameters of the two-*b* policies. As the agent has finite capacity *C*, $d_2$ is constrained by

10 $$\begin{aligned} C = \sum _{l=1}^{d_1} q_{d_1+d_2-l+1} b_1^l + b^{d_1} \sum _{l=1}^{d_2} q_{d_2-l+1} b_2^l \end{aligned}$$

and the optimal $b_1$, $b_2$, $d_1$ and *q* are found by

11 $$\begin{aligned} (b_1^*, b_2^*, d_1^*, q^*)= \mathop {{\mathrm{arg\,max}}}\limits _{b_1, b_2, d_1, q} V_{d_1, d_2, b_1, b_2, q}\;. \end{aligned}$$

What we showed so far are *heterogeneous* allocations where the $q_j$ can take arbitrary values as long as Eq. (10) is satisfied. As we did before, we will focus on the *homogeneous* subfamily of selective policies, where the sampling probability is one for all but for the last level, and $q_1$ is chosen such that the average capacity constraint in Eq. (10) is satisfied. After choosing the second branching factor $b_2$, the agent samples the following $d_2'$ levels as deep as they can until capacity is exhausted, namely $d_2'\equiv d_2(b_1, b_2, d_1, C)$ depends on both the branching factors, the switching depth and capacity. Defining the remaining samples at the last level $d_2'$ as $C_r=C-\left( \sum _{l=1}^{d_1} b_1^l + b^{d_1} \sum _{l=1}^{d_2'-1} b_2^l\right)$, then each of the $b_1^{d_1}b_2^{d_2'}$ nodes is given independently a sample with probability $q_1'= q_1(b_1, b_2, d_1, C)= C_r/(b_1^{d_1}b_2^{d_2'})$. The optimal policy is

12 $$\begin{aligned} (b_1^*, b_2^*, d_1^*)= \mathop {{\mathrm{arg\,max}}}\limits _{b_1, b_2, d_1} V_{d_1, d_2', b_1, b_2, q'}\;, \end{aligned}$$

where $V_{d_1, d_2, b_1, b_2, q}={\mathbb {E}}(J_d)$ is found by using the diffusion-maximization algorithm defined in Eqs. (4,9) and Eqs. (19,20,27,28) in Sect. “Value of exhaustive or selective search in a large tree with rational* p*” of the Methods.

### Discounted setting

We extend our algorithm by introducing a temporal discount factor $\gamma$, which exponentially reduces the value of rewards collected in deeper levels of the tree. A different –yet equivalent– interpretation of the temporal discount factor sees it as the *survival probability* for the agent to ‘live’ the next level and collect the future rewards. It follows that the closer the $\gamma$ is to one, the greater is the agent’s ability to foresee into the deeper levels. We rely on this alternative definition to generalize the diffusion-maximization algorithm for selective allocations described in Eq. (4,5) to the discounted case for $p=\frac{1}{2}$ and leave to Sect. “Value of exhaustive or selective search in a large tree with rational* p*” of the Methods the generalization to rational values. The generalized algorithm we show here incorporates the previous undiscounted one as the special case with $\gamma =1$. As before, we start from the last sampled level, and move backwards alternating diffusion and maximization steps. The introduction of the survival probability $\gamma$ has no effects on the last sampled level, as the agent is already myopic to future rewards due to the lack of resources. Consistently with what previously shown, the value of $J_1$ of a depth-1 tree is in the set $\{-1,0,1\}$. The action-value of $Q_1$ of each leaf has values $\{-1,0,1\}$ with probabilities $P(Q_1=1)=\frac{1}{2}q_1$, $P(Q_1 = 0) = 1-q_1$ and $P(Q_1=-1)=\frac{1}{2}q_1$. As *b* branches are available, the values of $J_1$ have probabilities $P(J_1=1) = 1- (1-\frac{q_1}{2})^b$, $P(J_1=0) =(1-\frac{q_1}{2})^b - (\frac{q_1}{2})^b$ and $P(J_1=-1) = (\frac{q_1}{2})^b$.

To compute $P(J_d)$ from $P(J_{d-1})$ we first relate $P(J_{d-1})$ with $P(Q_d)$ through the diffusion step. By definition, the action-value can be written as $Q_d = R_d + J_{d-1}$. In the discounted setting, with probability $1-\gamma$ the agent does not survive and hence they are not able to see the accumulated rewards in the previous steps $J_{d-1}$. It follows that, with probability $1-\gamma$, the only contribution to the action values comes from the immediate rewards $R_d$, while with probability $\gamma$ the agent will be able to see the contribution coming from $J_{d-1}$ (see scheme in Fig. 10a). The diffusion step takes then the form

13 $$\begin{aligned}&P(Q_{d}=d) = \frac{1}{2} q_d \gamma P(J_{d-1}=d-1)\nonumber \\&P(Q_{d}=d-1) = (1-q_d)\gamma P(J_{d-1}=d-1) + \frac{1}{2} q_d \gamma P(J_{d-1}=d-2)\nonumber \\&P(Q_{d}=d-2) = \frac{1}{2} q_d \gamma P(J_{d-1}=d-1) + (1-q_d) \gamma P(J_{d-1}=d-2) + \frac{1}{2} q_d \gamma P(J_{d-1}=d-3)\nonumber \\&\vdots \\&P(Q_{d}=1) = \frac{1}{2} q_d \gamma P(J_{d-1}=0) + (1-q_d) \gamma P(J_{d-1}=1) + \frac{1}{2} q_d \gamma P(J_{d-1}=2) + \frac{1}{2} q_d (1-\gamma )\nonumber \\&P(Q_{d}=0) = \frac{1}{2} q_d \gamma P(J_{d-1}=-1) + (1-q_d) \gamma P(J_{d-1}=0) + \frac{1}{2} q_d \gamma P(J_{d-1}=1) + (1-q_d) (1-\gamma )\nonumber \\&P(Q_{d}=-1) = \frac{1}{2} q_d \gamma P(J_{d-1}=-2) + (1-q_d) \gamma P(J_{d-1}=-1) + \frac{1}{2} q_d \gamma P(J_{d-1}=0) + \frac{1}{2} q_d (1-\gamma )\nonumber \\&\vdots \nonumber \\&P(Q_{d}=2-d) = \frac{1}{2} q_d \gamma P(J_{d-1}=1-d) + (1-q_d) \gamma P(J_{d-1}=2-d) + \frac{1}{2} q_d \gamma P(J_{d-1}=3-d) \nonumber \\&P(Q_{d}=1-d) = \frac{1}{2} q_d \gamma P(J_{d-1}=2-d) + (1-q_d)\gamma P(J_{d-1}=1-d)\nonumber \\&P(Q_{d}=-d) = \frac{1}{2} q_d\gamma P(J_{d-1}=1-d)\;,\nonumber \end{aligned}$$

where we can see the special contribution to the states $\{-1,0,1\}$ coming from the probability of ‘dying’, and the probability $\gamma$ rescaling all the $P(J_{d-1})$. The diffusion step is followed by the maximization step in Eq. (5). As before, iterating the diffusion and maximization steps in Eqs. (5, 13) with initial conditions $P(J_1)$ described above allows us to compute $V_{d,b,q,\gamma } = E(J_d)$, which is the value of playing a tree of depth *d*, branching factor *b*, per-level sampling probabilities *q* and survival probability $\gamma$. As the agent is limited by the finite sampling capacity described in Eq. (6), the optimal *b* and *q* are found by

14 $$\begin{aligned} (b^*,q^*) = \mathop {{\mathrm{arg\,max}}}\limits _{b,q} V_{d,b,q,\gamma } \;, \end{aligned}$$

subject to the capacity constraint in Eq. (6) and for a given $\gamma$.

### Value of exhaustive or selective search in a large tree with rational *p*

We extend our results for $p=\frac{1}{2}$ to the case of rational values $p=p_+=\frac{n}{n+1}$ and $p=p_+=\frac{1}{n+1}$ for any positive integer *n*. The zero-average reward constraint enforces that $p_++p_-=1$ and $p_+R_++p_-R_-=0$. We arbitrarily take $R_+=1$ and select $R_-$ so that the zero-average reward constraint is satisfied.

#### Reward probability $p=\frac{n}{n+1}$

We first consider $p=p_+=\frac{n}{n+1}$, which implies $p_-=\frac{1}{n+1}$. The zero-average constraint results in $R_-=-n$. We describe below how to compute the value of playing a large tree exhaustively and selectively with such a probability *p* of positive reward.

*Exhaustive allocation*. We begin by describing the value of a tree with one level ($d=1$), which will serve as initial condition for the diffusion-maximization algorithm. In this case, the cumulative reward can only be 1 or $-n$, that is, $J_1 \in \left\{ 1, -n \right\}$. Thus

$$\begin{aligned} P\left( J_1=1\right) =1-P\left( J_1=-n\right) =1-\left( \frac{1}{n+1}\right) ^b\;, \end{aligned}$$

where *b* is the number of branches.

As we have seen for $p=\frac{1}{2}$ in the main text, we can compute the probabilities for a tree of depth *d* starting from the probabilities of the cumulative reward of a tree of depth $d-1$ by alternating the *diffusion* and *maximization* steps. The diffusion step uses the probabilities of the cumulative reward $J_{d-1}$ of a tree of depth $d-1$ to compute the action values $Q_{d}$ of a tree of depth *d* using the possible rewards $R_d=\left\{ R_+=1,R_-=-n\right\}$. Both the cumulative reward $J_d$ and the action values $Q_d$ for a tree of depth *d* can take values $k=-nd + (n+1)i$, with $i\in \left\{ 0,1,2,\dots ,d\right\}$, where *i* is number of times the positive reward 1 was observed in the best possible path.

Using the above, the diffusion step becomes

15 $$\begin{aligned} \begin{aligned} P\left( Q_d=k\right) =\frac{1}{n+1}P\left( J_{d-1}=k+n\right) +\frac{n}{n+1}P\left( J_{d-1}=k-1\right) \;, \end{aligned} \end{aligned}$$

where it is understood that $P(J_{d-1}=k')=0$ if $k'$ lies outside the domain of $J_{d-1}$, in particular when $k'> d-1$ or $k'< -n(d-1)$, and thus some terms in the rhs of the above equation can become zero, by definition.

The maximization step is, as before,

16 $$\begin{aligned} P\left( J_d=k\right) =\left( P\left( Q_d\le k\right) \right) ^b-\left( P\left( Q_d\le k-1\right) \right) ^b\;. \end{aligned}$$

*Selective allocation*. The average finite capacity constraint enforces that

$$\begin{aligned} C=\sum _{l=1}^d q_{d-l+1}b^l\;, \end{aligned}$$

where $q_{d-l+1}$ is the sampling probability of tree level *l*. We underline the reverse order of the index of *q*, which is due to the fact that we are describing a backward algorithm: $q_1$ will appear in the first step and corresponds to the last level, $q_2$ in the second step and corresponds to the second last level, and so on. In selective allocation of samples, it is possible that a node is not sampled, and thus the possible values of both $J_d$ and $Q_d$ are

$$\begin{aligned} k=i - n j\;, \end{aligned}$$

with $i,j\in \{0, 1 \dots , d\}$ and $i+j\le d$, where *i* is the number of times the positive reward 1 is observed, and *j* is the number of times the negative reward $-n$ is observed.

We now proceed to compute the value of a tree with one level, and then use the diffusion-maximization algorithm to compute the value of a tree with any arbitrary depth *d*. The probabilities of the action values $Q_1$ for the branches of such a tree are

$$\begin{aligned} P\left( Q_1=-n\right)&=q_1p_-=\frac{q_1}{n+1}\\ P\left( Q_1=0\right)&=1-q_1\\ P\left( Q_1=1\right)&=q_1p_+=\frac{n q_1}{n+1}\;, \end{aligned}$$

and by using the maximization step, we obtain that the values $J_1$ take probabilities

$$\begin{aligned} P\left( J_1=-n\right)&=\left( P\left( Q_1\le -n\right) \right) ^b\\ P\left( J_1=0\right)&=\left( P\left( Q_1\le 0\right) \right) ^b- \left( P\left( Q_1\le -n\right) \right) ^b\\ P\left( J_1=1\right)&=\left( P\left( Q_1\le 1\right) \right) ^b- \left( P\left( Q_1\le 0\right) \right) ^b\;. \end{aligned}$$

Now, the diffusion step is

17 $$\begin{aligned} P\left( Q_d=k\right) =\left( 1-q_d\right) P\left( J_{d-1}=k\right) + \frac{1}{n+1}q_dP\left( J_{d-1}=k + n\right) + \frac{n}{n+1}q_dP\left( J_{d-1}=k -1\right) \;, \end{aligned}$$

where, again, it is understood that $P(J_{d-1}=k')=0$ when $k'$ lies outside the domain of $J_{d-1}$, in particular when $k'> d-1$ or $k'< -n (d-1)$, and thus many terms contribute zero.

The diffusion step is then followed by the usual maximization step

18 $$\begin{aligned} P\left( J_d=k\right) =\left( P\left( Q_d\le k\right) \right) ^b-\left( P\left( Q_d\le k-1\right) \right) ^b\;. \end{aligned}$$

*Two-b* *selective allocation*. As we sample $d_1\ge 1$ levels with branching factor $b_1$ and $d_2\ge 1$ with $b_2$, the average finite capacity constraint takes the form

$$\begin{aligned} C= \sum _{l=1}^{d_1}q_{d_1+d_2-l+1}b_1^l + b_1^{d_1}\sum _{l=1}^{d_2}q_{d_2-l+1} b_2^l\;, \end{aligned}$$

where again we note the reverse index order for *q*. To compute the value of a tree with arbitrary depth $d_1+d_2$, we start by computing the value of a tree of one level. The probabilities of action values $Q_1$ of such a tree are the same as before

$$\begin{aligned} P\left( Q_1=-n\right)&=q_1p_-=\frac{q_1}{n+1}\\ P\left( Q_1=0\right)&=1-q_1\\ P\left( Q_1=1\right)&=q_1p_+=\frac{n q_1}{n+1}\;. \end{aligned}$$

We use then the maximization step to choose the best out of $b_2$ options

$$\begin{aligned} P\left( J_1=-n\right)&=\left( P\left( Q_1\le -n\right) \right) ^{b_2}\\ P\left( J_1=0\right)&=\left( P\left( Q_1\le 0\right) \right) ^{b_2}- \left( P\left( Q_1\le -n\right) \right) ^{b_2}\\ P\left( J_1=1\right)&=\left( P\left( Q_1\le 1\right) \right) ^{b_2}- \left( P\left( Q_1\le 0\right) \right) ^{b_2}\;. \end{aligned}$$

With the values of $J_1$, we construct the value of playing a tree with arbitrary depth *d* by iterating the diffusion-maximization algorithm. The diffusion step is, as before

19 $$\begin{aligned} P\left( Q_d=k\right) =\left( 1-q_d\right) P\left( J_{d-1}=k\right) + \frac{1}{n+1}q_dP\left( J_{d-1}=k + n\right) + \frac{n}{n+1}q_dP\left( J_{d-1}=k -1\right) \;, \end{aligned}$$

where, again, it is understood that $P(J_{d-1}=k')=0$ when $k'$ lies outside the domain of $J_{d-1}$. The maximization step here takes the form

20 $$\begin{aligned} P\left( J_d=k\right) =\left( P\left( Q_d\le k\right) \right) ^{b_j}-\left( P\left( Q_d\le k-1\right) \right) ^{b_j}\;. \end{aligned}$$

with $b_j=b_2$ if $d\le d_2$ or $b_j=b_1$ if $d>d_2$. Once more, we remark the backward nature of the algorithms we are describing, facing first the $d_2$ levels with branching $b_2$.

*Discounted selective allocation.* As shown in the probability $p=\frac{1}{2}$ case, the introduction of a discount factor does not affect the probabilities of the action-values of a tree of one level, that are

$$\begin{aligned} P\left( Q_1=-n\right)&=q_1p_-=\frac{q_1}{n+1}\\ P\left( Q_1=0\right)&=1-q_1\\ P\left( Q_1=1\right)&=q_1p_+=\frac{n q_1}{n+1}\;, \end{aligned}$$

By using the maximization step, we obtain the probabilities of the $J_1$ values as

$$\begin{aligned} P\left( J_1=-n\right)&=\left( P\left( Q_1\le -n\right) \right) ^b\\ P\left( J_1=0\right)&=\left( P\left( Q_1\le 0\right) \right) ^b- \left( P\left( Q_1\le -n\right) \right) ^b\\ P\left( J_1=1\right)&=\left( P\left( Q_1\le 1\right) \right) ^b- \left( P\left( Q_1\le 0\right) \right) ^b\;. \end{aligned}$$

Moving to deeper trees, we introduce the survival probability $\gamma$, and the consequent additional contribution to the states $\{-n,0,1\}$ coming from the probability of ‘dying’ and collecting uniquely the immediate reward $R_d$. Hence, the diffusion step takes the form

21 $$\begin{aligned} P\left( Q_d=k\right)= & {} \left( 1-q_d\right) \gamma P\left( J_{d-1}=k\right) + \frac{1}{n+1}q_d\gamma P\left( J_{d-1}=k + n\right) + \frac{n}{n+1}q_d \gamma P\left( J_{d-1}=k -1\right) \nonumber \\ P\left( Q_d=-n\right)= & {} \left( 1-q_d\right) \gamma P\left( J_{d-1}=-n\right) + \frac{1}{n+1}q_d\gamma P\left( J_{d-1}= 0 \right) + \frac{n }{n+1} q_d\gamma P\left( J_{d-1}=-n-1\right) \nonumber \\&+ \frac{1}{n+1} q_d (1-\gamma )\nonumber \\ P\left( Q_d=0\right)= & {} \left( 1-q_d\right) \gamma P\left( J_{d-1}=0\right) + \frac{1}{n+1}q_d\gamma P\left( J_{d-1}= n \right) + \frac{n}{n+1}q_d\gamma P\left( J_{d-1}=-1\right) \\&+(1-q_d) (1-\gamma )\nonumber \\ P\left( Q_d=1\right)= & {} \left( 1-q_d\right) \gamma P\left( J_{d-1}=1\right) + \frac{1}{n+1}q_d \gamma P\left( J_{d-1}= 1+n \right) + \frac{n}{n+1}q_d \gamma P\left( J_{d-1}=0 \right) \nonumber \\&+\frac{n}{n+1}q_d(1-\gamma ) \;, \nonumber \end{aligned}$$

where, again, it is understood that $P(J_{d-1}=k')=0$ when $k'$ lies outside the domain of $J_{d-1}$, in particular when $k'> d-1$ or $k'< -n (d-1)$, and thus many terms contribute zero. The diffusion step is then followed by the usual maximization step

22 $$\begin{aligned} P\left( J_d=k\right) =\left( P\left( Q_d\le k\right) \right) ^b-\left( P\left( Q_d\le k-1\right) \right) ^b\;. \end{aligned}$$

#### Algorithmic complexity

The complexity of the algorithm is proportional to the number of equations, which equals the sum of the number of possible different states per level. As we said above, the possible state values $J_s$ at level *s* are $k=i-nj$, with $i,j \ge 0$ and $i+j \le s$. As *n* is an integer, it is possible to have repeated values of *k* for different values of *i* and *j* within the allowed set.

To count the number of distinct states, we start by noticing that if $j=0$, then $k=i$, and thus there are $s+1$ distinct states (Fig. 3a, orange points in the bottom row of the triangle). Assume first that $s<n$. If $j=1$, then $k=i-n$, where *i* lies between 0 and $s-1$ (second bottom row of points in the triangle). As $s<n$, the resulting states $k=i-n$ do not reach $k=0$, and thus all of them are distinct from those corresponding to the bottom row. If $j=2$, the states are $k=i-2n$, where *i* lies between 0 and $s-2$ (third bottom row), and as the values of *k* do not reach $-n$, the new states are all new. In conclusion if $s<n$ the total number of distinct states *N*(*n*, *s*) in level *s* is

$$\begin{aligned} N(n,s) = \frac{(s+1)(s+2)}{2}\;, \end{aligned}$$

For $s \ge n$, there are many values of *i* and *j* that result in repeated states *k* (Fig. 3b, violet points). If $j=0$, then $k=i$, resulting in $s+1$ distinct states, as before (orange points in the bottom row of the triangle). If $j=1$, then $k=i-n$, resulting in the states $\{-n,n+1,\ldots ,0,\ldots ,s-n\}$, of which all states equal or above 0 are repeated (violet points in the second bottom row). Thus, there are *n* new states. Extending the above, for each *j* in $\{1,\ldots ,n\}$ there are *n* new states, and for larger values of *j* the new states are $s-j+1$.

In conclusion, if $s \ge n$ the total number of distinct states *N*(*n*, *s*) in level *s* is

$$\begin{aligned} N(n,s) = (n+1)s - \frac{n(n-1)}{2} + 1\;, \end{aligned}$$

From here, the scaling of states is proportional to the level *s*, and for large *s* the term *ns* dominates. Therefore, when summing up distinct states from the first to the last level *d* of the tree, we conclude that the complexity of the maximization-diffusion algorithm is ${\mathcal {O}}\left( n d^2 b\right)$, where we take into account that for every state we need to perform a maximization step (a power operation that counts *b* per state). Analogous steps can be made for the case considered next of $p=\frac{1}{n+1}$ to reach to an identical algorithmic complexity.

#### Reward probability $p=\frac{1}{n+1}$

We proceed by considering $p=p_+=\frac{1}{n+1}$ which implies $p_-=\frac{n}{n+1}$. The zero-average reward leads in this case to a negative reward $R_-=-\frac{1}{n}$. We show here how to compute the value of playing a large tree, exhaustively and selectively, and with such reward probability $p_+$.

*Exhaustive allocation*. As shown before, the initial conditions for the diffusion-maximization algorithm come from the value of a tree with just one level $(d=1)$. For a single level tree the cumulative reward can only be 1 or $-\frac{1}{n}$, namely $J_1\in \{1,-\frac{1}{n}\}$. Thus, for a number *b* of branches

$$\begin{aligned} P\left( J_1=1\right) =1- P\left( J_1=-\frac{1}{n}\right) =1-\left( \frac{n}{n+1}\right) ^b\;. \end{aligned}$$

Again we can compute the probabilities of $J_d$ for a tree of depth *d* from the probabilities of $J_{d-1}$ for a tree of depth $d-1$ using *diffusion*-*maximization*. In the diffusion step, we use the probabilities of $J_{d-1}$ of a tree of depth $d-1$ to compute the action values $Q_d$ of the tree of depth *d* along with the possible rewards $R_d=\{R_+=1, R_-=-\frac{1}{n}\}$. For a tree of depth *d*, both the cumulative reward $J_d$ and the action value $Q_d$ can take the values $k=-\frac{d}{n}+\left( \frac{1}{n}+1\right) i$ with $i\in \{0,1,\dots ,d\}$, where *i* is the number of times that the positive reward $R_+=1$ is observed.

Now, the diffusion step becomes

23 $$\begin{aligned} P\left( Q_d=k\right) =\frac{n}{n+1}P\left( J_{d-1}=k+\frac{1}{n}\right) +\frac{1}{n+1}P\left( J_{d-1}=k-1\right) \;, \end{aligned}$$

where again the probabilities $P(J_{d-1}=k')$ are zero when $k'$ lies outside the domain of $J_{d-1}$, in particular when $k'> d-1$ or $k'<-\frac{d-1}{n}$.

After the diffusion, the maximization step is always

24 $$\begin{aligned} P\left( J_d=k\right) =\left( P\left( Q_d\le k\right) \right) ^b -\left( P\left( Q_d\le k-1\right) \right) ^b\;. \end{aligned}$$

*Selective allocation*. As we have shown in the main text for $p=\frac{1}{2}$, and previously here for $p=\frac{n}{n+1}$, in selective allocation we consider the average finite capacity constraint

$$\begin{aligned} C=\sum _{l=1}^d q_{d-l+1}b^l\;, \end{aligned}$$

where $q_{d-l+1}$ is the sampling probability of tree level *l*. As nodes might not be sampled, the possible values of both $J_d$ and $Q_d$ are

$$\begin{aligned} k = i-\frac{j}{n}\;, \end{aligned}$$

with $i,j\in \{0,1,\dots , d\}$ and $i+j\le d$, where *i* is the number of times that the positive reward 1 is observed, and *j* is the number of times that the the negative reward $-\frac{1}{n}$ is observed in the best possible path. We first compute the value of a tree with depth 1 and then use the diffusion-maximization algorithm to perform induction over *d*. The probabilities of the action values $Q_1$ for the branches of a tree with $d=1$ are

$$\begin{aligned} P\left( Q_1=-\frac{1}{n}\right)&=q_1p_-=\frac{nq_1}{n+1}\\ P\left( Q_1=0\right)&=\left( 1-q_1\right) \\ P\left( Q_1=1\right)&=q_1p_+=\frac{q_1}{n+1}\;.\\ \end{aligned}$$

Thus, the probability of $J_1$ are obtained by using the maximization step

$$\begin{aligned} P\left( J_1=-\frac{1}{n}\right)&=\left( P\left( Q_1\le -\frac{1}{n}\right) \right) ^b\\ P\left( J_1=0\right)&=\left( P\left( Q_1\le 0\right) \right) ^b- \left( P\left( Q_1\le -\frac{1}{n}\right) \right) ^b\\ P\left( J_1=1\right)&=\left( P\left( Q_1\le 1\right) \right) ^b- \left( P\left( Q_1\le 0\right) \right) ^b\;. \end{aligned}$$

Given these initial conditions, it is easy to see that the diffusion step for level *d* is

25 $$\begin{aligned} P\left( Q_d=k\right) =\left( 1-q_d\right) P\left( J_{d-1}=k\right) + \frac{n}{n+1}q_dP\left( J_{d-1}=k+\frac{1}{n}\right) + \frac{1}{n+1}q_dP\left( J_{d-1}=k-1\right) \;, \end{aligned}$$

where again it is understood that $P(J_{d-1}=k')=0$ when $k'$ lies outside the domain of $J_{d-1}$.

The diffusion step is then followed by the usual maximization step

26 $$\begin{aligned} P\left( J_d=k\right) =\left( P\left( Q_d\le k\right) \right) ^b-\left( P\left( Q_d\le k-1\right) \right) ^b\;. \end{aligned}$$

*Two*-*b* *allocation*. As shown before, when two branching factors are considered, the average finite capacity constraint takes the form

$$\begin{aligned} C= \sum _{l=1}^{d_1}q_{d_1+d_2-l+1}b_1^l + b_1^{d_1}\sum _{l=1}^{d_2}q_{d_2-l+1} b_2^l\;. \end{aligned}$$

The value of playing a tree of depth *d* can be computed by iterating the diffusion-maximization algorithm starting from a 1-level tree. For a tree with $d=1$, the probabilities of the action values $Q_1$ are

$$\begin{aligned} P\left( Q_1=-\frac{1}{n}\right)&=q_1p_-=\frac{nq_1}{n+1}\\ P\left( Q_1=0\right)&=\left( 1-q_1\right) \\ P\left( Q_1=1\right)&=q_1p_+=\frac{q_1}{n+1}\;, \end{aligned}$$

from which we obtain the values of $J_1$ with the maximization step

$$\begin{aligned} P\left( J_1=-\frac{1}{n}\right)&=\left( P\left( Q_1\le -\frac{1}{n}\right) \right) ^{b_2}\\ P\left( J_1=0\right)&=\left( P\left( Q_1\le 0\right) \right) ^{b_2}- \left( P\left( Q_1\le -\frac{1}{n}\right) \right) ^{b_2}\\ P\left( J_1=1\right)&=\left( P\left( Q_1\le 1\right) \right) ^{b_2}- \left( P\left( Q_1\le 0\right) \right) ^{b_2}\;. \end{aligned}$$

The diffusion step for the generic level *d* then takes the form

27 $$\begin{aligned} P\left( Q_d=k\right) =\left( 1-q_d\right) P\left( J_{d-1}=k\right) + \frac{n}{n+1}q_dP\left( J_{d-1}=k+\frac{1}{n}\right) + \frac{1}{n+1}q_dP\left( J_{d-1}=k-1\right) \;, \end{aligned}$$

with $P(J_{d-1}=k')=0$ when $k'$ lies outside the domain of $J_{d-1}$. The diffusion step is followed by the maximization step

28 $$\begin{aligned} P\left( J_d=k\right) =\left( P\left( Q_d\le k\right) \right) ^{b_j}-\left( P\left( Q_d\le k-1\right) \right) ^{b_j}\;, \end{aligned}$$

where, again, in the described backward algorithm $b_j=b_2$ if $d\le d_2$ or $b_j=b_1$ if $d> d_2$.

*Discounted selective allocation.* The value of playing a tree of depth *d* can be computed by iterating the diffusion-maximization algorithm starting from a 1-level tree. For a tree with $d=1$, the discount factor does not play any role, therefore the probabilities of the action values $Q_1$ are

$$\begin{aligned} P\left( Q_1=-\frac{1}{n}\right)&=q_1p_-=\frac{nq_1}{n+1}\\ P\left( Q_1=0\right)&=\left( 1-q_1\right) \\ P\left( Q_1=1\right)&=q_1p_+=\frac{q_1}{n+1}\;, \end{aligned}$$

from which we obtain the values of $J_1$ with the maximization step

$$\begin{aligned} P\left( J_1=-\frac{1}{n}\right)&=\left( P\left( Q_1\le -\frac{1}{n}\right) \right) ^{b}\\ P\left( J_1=0\right)&=\left( P\left( Q_1\le 0\right) \right) ^{b}- \left( P\left( Q_1\le -\frac{1}{n}\right) \right) ^{b}\\ P\left( J_1=1\right)&=\left( P\left( Q_1\le 1\right) \right) ^{b}- \left( P\left( Q_1\le 0\right) \right) ^{b}\;. \end{aligned}$$

As shown before, when we move to $d>1$ in the discounted setting we have to consider the special contribution coming from the probability of ‘dying’ to the states $\left\{ -\frac{1}{n},0,1\right\}$. It follows that the diffusion step takes the form

29 $$\begin{aligned} P\left( Q_d=k\right)= & {} \left( 1-q_d\right) \gamma P\left( J_{d-1}=k\right) + \frac{n}{n+1}q_d\gamma P\left( J_{d-1}=k+\frac{1}{n}\right) + \frac{1}{n+1}q_d\gamma P\left( J_{d-1}=k-1\right) \nonumber \\ P\left( Q_d=-\frac{1}{n}\right)= & {} \left( 1-q_d\right) \gamma P\left( J_{d-1}=-\frac{1}{n}\right) +\frac{n}{n+1}q_d\gamma P\left( J_{d-1}=0\right) +\nonumber \\&+\frac{1}{n+1}q_d\gamma P\left( J_{d-1}=-\frac{1}{n}-1\right) + \frac{n}{n+1} q_d(1-\gamma ) \\ P\left( Q_d=0 \right)= & {} \left( 1-q_d\right) \gamma P\left( J_{d-1}=0\right) + \frac{n}{n+1}q_d\gamma P\left( J_{d-1}=\frac{1}{n}\right) + \frac{1}{n+1}q_d\gamma P\left( J_{d-1}=-1\right) + \nonumber \\&+ (1-q_d)(1-\gamma )\nonumber \\ P\left( Q_d=1\right)= & {} \left( 1-q_d\right) \gamma P\left( J_{d-1}=1\right) + \frac{n}{n+1}q_d\gamma P\left( J_{d-1}=1+\frac{1}{n}\right) + \frac{1}{n+1}q_d\gamma P\left( J_{d-1}=0\right) \nonumber \\&+ \frac{1}{n+1} q_d(1-\gamma )\;,\nonumber \end{aligned}$$

where again it is understood that $P(J_{d-1}=k')=0$ when $k'$ lies outside the domain of $J_{d-1}$.

The diffusion step is then followed by the usual maximization step

30 $$\begin{aligned} P\left( J_d=k\right) =\left( P\left( Q_d\le k\right) \right) ^b-\left( P\left( Q_d\le k-1\right) \right) ^b\;. \end{aligned}$$

## Data Availability

The data generating the results and the C codes to reproduce them, as well as the Matlab codes to generate figures, are available at this public GitHub repository.

## References

[1] Russell S, Wefald E (1991). Principles of metareasoning. Artif. Intell..

[2] Gershman SJ, Horvitz EJ, Tenenbaum JB (2015). Computational rationality: A converging paradigm for intelligence in brains, minds, and machines. Science.

[3] Griffiths TL, Lieder F, Goodman ND (2015). Rational use of cognitive resources: Levels of analysis between the computational and the algorithmic. Top. Cogn. Sci..

[4] Moreno-Bote R, Ramírez-Ruiz J, Drugowitsch J, Hayden BY (2020). Heuristics and optimal solutions to the breadth-depth dilemma. Proc. Natl. Acad. Sci..

[5] Patel, N., Acerbi, L. & Pouget, A. Dynamic allocation of limited memory resources in reinforcement learning. arXiv:2011.06387 (2020).

[6] Malloy, T., Sims, C. R., Klinger, T., Liu, M., Riemer, M. & Tesauro, G. Deep RL With Information Constrained Policies: Generalization in Continuous Control. arXiv:2010.04646 (2020).

[7] Horowitz E, Sahni S (1978). Fundamentals of Computer Algorithms.

[8] Korf RE (1985). Depth-first iterative-deepening. Artif. Intell..

[9] Miller DP (1981). The depth/breadth tradeoff in hierarchical computer menus. Proc. Human Factors Soc. Annu. Meet..

[10] Ramirez-Ruiz, J. & Moreno-Bote, R. Optimal allocation of finite sampling capacity in accumulator models of multi-alternative decision making. *Cognitive Science* **46**, (2022). 10.1111/cogs.13143 PMC9285422 35523123

[11] Vidal, A., Soto-Faraco, S. & Moreno-Bote, R. Humans balance breadth and depth: Near-optimal performance in many-alternative decision making. PsyArXiv (2021).

[12] Turner SF, Bettis RA, Burton RM (2002). Exploring depth versus breadth in knowledge management strategies. Comput. Math. Organ. Theory.

[13] Schwartz MS, Sadler PM, Sonnert G, Tai RH (2009). Depth versus breadth: How content coverage in high school science courses relates to later success in college science coursework: Depth versus breadth. Sci. Educ..

[14] Sutton RS, Barto AG (1998). Reinforcement learning: An introduction. Adaptive Computation and Machine Learning.

[15] Browne CB, Powley E, Whitehouse D, Lucas SM, Cowling PI, Rohlfshagen P, Tavener S, Perez D, Samothrakis S, Colton S (2012). A survey of Monte Carlo tree search methods. IEEE Trans. Comput. Intell. AI Games.

[16] Berry DA, Chen RW, Zame A, Heath DC, Shepp LA (1997). Bandit problems With infinitely many arms. The Annals of Statistic.

[17] Wang W, Audibert J, Munos R, Koller D, Schuurmans D, Bengio Y, Bottou L (2009). Algorithms for infinitely many-armed bandits. Advances in Neural Information Processing Systems.

[18] Callaway, F., van Opheusden, B., Gul, S., Das, P., Krueger, P., Lieder, F. & Griffiths, T. Human planning as optimal information seeking. PsyArXiv (2021).

[19] Hay, N., Russell, S., Tolpin, D. & Shimony, S. E. Selecting computations: Theory and applications, arXiv:1408.2048 (2014).

[20] Sezener, E. & Dayan, P. Static and dynamic values of computation in mcts. In *Proceedings of the 36th Conference on Uncertainty in Artificial Intelligence (UAI)*, Proceedings of Machine Learning Research, 205–220. (PMLR, 2020).

[21] Chen, W., Hu, W., Li, F., Li, J., Liu, Y. & Lu, P. Combinatorial multi-armed bandit with general reward functions. arXiv:1610.06603 (2018).

[22] Simon HA, McGuire CB, Radner R (1972). Theories of bounded rationality. Decision and Organization.

[23] Evans JSBT (2006). The heuristic-analytic theory of reasoning: Extension and evaluation. Psychonom. Bull. Rev..

[24] Nanay B (2016). The role of imagination in decision-making. Mind Lang..

[25] Tversky A, Kahneman D (1973). Availability: A heuristic for judging frequency and probability. Cogn. Psychol..

[26] Tversky A (1972). Elimination by aspects: A theory of choice. Psychol. Rev..

[27] Pezzulo G (2008). Coordinating with the future: The anticipatory nature of representation. Mind. Mach..

[28] Ratcliff R, Murdock BB (1976). Retrieval processes in recognition memory. Psychol. Rev..

[29] Shadlen MN, Shohamy D (2016). Decision making and sequential sampling from memory. Neuron.

[30] Coulom R, van den Herik HJ, Ciancarini P, Donkers HHLMJ (2007). Efficient selectivity and backup operators in Monte-Carlo tree search. Computers and Games.

[31] Silver D, Schrittwieser J, Simonyan K, Antonoglou I, Huang A, Guez A, Hubert T, Baker L, Lai M, Bolton A, Chen Y, Lillicrap T, Hui F, Sifre L, van den Driessche G, Graepel T, Hassabis D (2017). A general reinforcement learning algorithm that masters chess, shogi, and go through self-play. Nature.

[32] Kaelbling LP, Littman ML, Moore AW (1996). Reinforcement learning: A survey. J. Artif. Int. Res..

[33] Clark A, Grush R (1999). Towards a cognitive robotics. Adapt. Behav..

[34] Grush R (2004). The emulation theory of representation: Motor control, imagery, and perception. Behav. Brain Sci..

[35] Doll BB, Simon DA, Daw ND (2012). The ubiquity of model-based reinforcement learning. Curr. Opin. Neurobiol..

[36] Simons JS, Garrison JR, Johnson MK (2017). Brain mechanisms of reality monitoring. Trends Cogn. Sci..

[37] Hamrick JB (2019). Analogues of mental simulation and imagination in deep learning. Curr. Opin. Behav. Sci..

[38] Gupta AS, van der Meer MAA, Touretzky DS, Redish AD (2010). Hippocampal replay is not a simple function of experience. Neuron.

[39] Pfeiffer BE, Foster DJ (2013). Hippocampal place-cell sequences depict future paths to remembered goals. Nature.

[40] Hauser JR, Wernerfelt B (1990). An evaluation cost model of consideration sets. J. Consum. Res..

[41] Stigler GJ (1961). The Economics of Information. J. Polit. Econ..

[42] Roberts JH, Lattin JM (1991). Development and testing of a model of consideration set composition. J. Mark. Res..

[43] Mehta N, Rajiv S, Srinivasan K (2003). Price uncertainty and consumer search: A structural model of consideration set formation. Mark. Sci..

[44] De los Santos B, Hortaçsu A, Wildenbeest MR (2012). Testing models of consumer search using data on web browsing and purchasing behavior. Am. Econ. Rev..

[45] Scheibehenne B, Greifeneder R, Todd PM (2010). Can there ever be too many options? A meta-analytic review of choice overload. J. Consum. Res..

[46] Keramati M, Smittenaar P, Dolan RJ, Dayan P (2016). Adaptive integration of habits into depth-limited planning defines a habitual-goal-directed spectrum. Proc. Natl. Acad. Sci..

[47] Hamrick, J. B., Ballard, A. J., Pascanu, R., Vinyals, O., Heess, N. & Battaglia, P. W. Metacontrol for Adaptive Imagination-Based Optimization. arXiv:1705.02670 (2017).

[48] Pascanu, R., Li, Y., Vinyals, O., Heess, N., Buesing, L., Racanière, S., Reichert, D., Weber, T., Wierstra, D. & Battaglia, P. Learning model-based planning from scratch. arXiv:1707.06170 (2017).

[49] Weber, T., Racanière, S., Reichert, D. P., Buesing, L., Guez, A., Rezende, D. J., Badia, A. P., Vinyals, O., Heess, N., Li, Y., Pascanu, R., Battaglia, P., Hassabis, D., Silver, D. & Wierstra, D. Imagination-Augmented Agents for Deep Reinforcement Learning. arXiv:1707.06203 (2018).

[50] Hafner, D., Lillicrap, T., Ba, J. & Norouzi, M. Dream to Control: Learning Behaviors by Latent Imagination. biorXiv (2020).

[51] Pearl J, Korf RE (1987). Search techniques. Annu. Rev. Comput. Sci..

[52] Sezener CE, Dezfouli A, Keramati M (2019). Optimizing the depth and the direction of prospective planning using information values. PLOS Comput. Biol..

[53] Zylberberg A (2022). Decision prioritization and causal reasoning in decision hierarchies. PLoS Comput. Biol..

[54] Snider J, Lee D, Poizner H, Gepshtein S (2015). Prospective optimization with limited resources. PLoS Comput. Biol..

[55] Wu, C. M., Schulz, E., Speekenbrink, M., Nelson, J. D. & Meder, B. Mapping the unknown: The spatially correlated multi-armed bandit . In Gunzelmann, G., Howes, A., Tenbrink, T. & Davelaar, E. editors, *Proceedings of the 39th Annual Meeting of the Cognitive Science Society*, 1357–1362 (Austin, TX, 2017).

[56] Gupta S, Chaudhari S, Joshi G, Yagan O (2021). Multi-armed bandits with correlated arms. IEEE Trans. Inf. Theory.

[57] Tolpin D, Shimony S (2021). MCTS based on simple regret. Proc. AAAI Conf. Artif. Intell..

[58] Gold JI, Shadlen MN (2007). The neural basis of decision making. Annu. Rev. Neurosci..

[59] Churchland AK, Kiani R, Shadlen MN (2008). Decision-making with multiple alternatives. Nat. Neurosci..

[60] Drugowitsch J, Moreno-Bote R, Churchland AK, Shadlen MN, Pouget A (2012). The cost of accumulating evidence in perceptual decision making. J. Neurosci..

[61] Krajbich I, Armel C, Rangel A (2010). Visual fixations and the computation and comparison of value in simple choice. Nat. Neurosci..

[62] Krusche, M. J. F., Schulz, E., Guez, A. & Speekenbrink, M. Adaptive planning in human search. biorXiv (2018).

[63] Hayden BY, Moreno-Bote R (2018). A neuronal theory of sequential economic choice. Brain Neurosci. Adv..

